# A multicenter randomized trial to assess the efficacy of CONvalescent plasma therapy in patients with Invasive COVID-19 and acute respiratory failure treated with mechanical ventilation: the CONFIDENT trial protocol

**DOI:** 10.1186/s12890-020-01361-x

**Published:** 2020-12-07

**Authors:** Benoît Misset, Eric Hoste, Anne-Françoise Donneau, David Grimaldi, Geert Meyfroidt, Michel Moutschen, Veerle Compernolle, André Gothot, Daniel Desmecht, Mutien Garigliany, Tome Najdovski, Pierre-François Laterre

**Affiliations:** 1 Department of Intensive Care Medicine, Liege University Hospital, Domaine Universitaire du Sart Tilman, 4020 Liège, Belgium; 2 Department of Intensive Care Medicine, Gent University Hospital, Gent, Belgium; 3 Biostatistic Unit, Public Health Department, Liège University, Liège, Belgium; 4 Department of Intensive Care Medicine, Cliniques Universitaires de Bruxelles - Erasme, Université Libre de Bruxelles, Brussels, Belgium; 5 Department of Intensive Care Medicine, University Hospitals Leuven, Leuven, Belgium; 6 Department of Infectious Diseases, Liege University Hospital, Liège, Belgium; 7 Blood Services from the Red Cross, Mechelen, Belgium; 8 Department of Immunohematology, Liege University Hospital, Liège, Belgium; 9 Department of Animal Pathology, Liège University, Liège, Belgium; 10 Blood Services From the Red Cross, Suarlée, Belgium; 11 Department of Intensive Care Medicine, Saint-Luc University Hospital, Brussels, Belgium

**Keywords:** COVID-19, Convalescent plasma, Randomized, Respiratory failure, Mechanical ventilation

## Abstract

**Background:**

The COVID-19 pandemic reached Europe in early 2020. Convalescent plasma is used without a consistent evidence of efficacy. Our hypothesis is that passive immunization with plasma collected from patients having contracted COVID-19 and developed specific neutralizing antibodies may alleviate symptoms and reduce mortality in patients treated with mechanical ventilation for severe respiratory failure during the evolution of SARS-CoV-2 pneumonia.

**Methods:**

We plan to include 500 adult patients, hospitalized in 16 Belgian intensive care units between September 2020 and 2022, diagnosed with SARS-CoV-2 pneumonia, under mechanical ventilation for less than 5 days and a clinical frailty scale less than 6. The study treatment will be compared to standard of care and allocated by randomization in a 1 to 1 ratio without blinding. The main endpoint will be mortality at day 28. We will perform an intention to treat analysis. The number of patients to include is based on an expected mortality rate at day 28 of 40 percent and an expected relative reduction with study intervention of 30 percent with α risk of 5 percent and β risk of 20 percent.

**Discussion:**

This study will assess the efficacy of plasma in the population of mechanically ventilated patients. A stratification on the delay from mechanical ventilation and inclusion will allow to approach the optimal time use. Selecting convalescent plasmas with a high titer of neutralizing antibodies against SARS-CoV-2 will allow a homogeneous study treatment. The inclusion in the study is based on the consent of the patient or his/her legal representative, and the approval of the Investigational Review Board of the University hospital of Liège, Belgium. A data safety monitoring board (DSMB) has been implemented. Interim analyses have been planned at 100, 2002, 300 and 400 inclusions in order to decide whether the trail should be discontinued prematurely for ethical issues. We plan to publish our results in a peer-reviewed journal and to present them at national and international conferences.

**Funding and registration:**

The trial is funded by the Belgian Health Care Knowledge Center KCE # COV201004

**Trial registration:**

Clinicaltrials.gov registration number NCT04558476. Registered 14 September 2020—Retrospectively registered, https://clinicaltrials.gov/ct2/show/NCT04558476

## Strengths and limitations of this study

1. This study will be the first to assess the efficacy of plasma collected from convalescents of COVID-19 in the population of mechanically ventilated patients. The high mortality of this specific population increases the chance to observe a difference between the two groups.
2. The prospective and randomized design will allow to give a solid answer to the question. A stratification on the delay from mechanical ventilation and inclusion in the study will allow to determine the optimal time to use the convalescent plasma
3. The participation of 16 centers of all Belgium will insure the external validity of the results and the recruitment of the patients in an acceptable time period
4. Selecting convalescent plasmas with a high titer of neutralizing antibodies against SARS-CoV-2 will guaranty an homogeneous treatment in the intervention group
5. Planning iterative interim analyses will allow to stop the trial early in case of an ethical issue induced by the observed results

## Background

The COVID-19 pandemic started in China in December 2019 and spread to European countries in early 2020, affecting most severely Italy, Spain and France. Belgium was affected during the same wave and first deaths due to COVID-19 were reported in early March 2020. As of October 2020, more than 30 million cases, including 1 million deaths, have been confirmed over the world and almost 90,000 cases, including 9900 deaths, have been declared in Belgium [1]. The potential duration of the current pandemic is largely unknown and epidemics with coronaviruses are likely to recur.

Current data from the Belgian surveillance system indicate that 20% of the hospitalized patients are in ICUs [2]. Thirty to sixty percent of the mechanically ventilated patients are likely to die [3, 4]. A race for developing active therapies and vaccines started since the onset of the pandemic [5]. Therapy with plasma collected from convalescent patients has been proposed to provide passive immunization to the patients at risk of developing severe COVD-19 in preliminary studies [6, 7] and in a randomized controlled trial that ended prematurely due to the end of the contaminations in China [8]. In this series, 103 patients were randomized and a subgroup analysis indicated that convalescent plasma was associated with a 30% relative reduction of mortality in the severe patients [8]. A direct antiviral effect of neutralizing antibodies within the convalescent plasma is likely to play a central role in the beneficial effect of the treatment. Two consecutive surveys in USA indicated that the use of convalescent plasma appears safe. Serious adverse events attributed to the plasma were estimated to be around 0.02 percent in 5,000 patients and mortality was estimated to be 41% at day 30 in those 27 percent of the patients who were under mechanical ventilation [9, 10]. In this series, the titer of antibodies measured in the convalescent plasma was correlated to the survival of the patient [10]. In a series of 175 patients with mild COVID-19 from Shangaï, China, Wu et al. [11] observed that the titers of neutralizing antibodies assessed at hospital discharge to SARS-CoV-2 varied substantially. In 11 patients with sequential assessments, high titers over 1/256 were measured as early as 10 to 16 days of disease onset.

As of October 6, 2020, 71 randomized controlled trials have been posted on Clinicaltrials.gov. Eighteen trials plan to enroll at least 300 patients. This study is the only one to include specifically mechanical ventilated patients.

Our hypothesis is that passive immunization with plasma collected from patients having contracted COVID-19 and developed specific antibodies may alleviate symptoms and viral load of SARS-CoV-2 and reduce mortality in patients treated with mechanical ventilation for severe respiratory failure during the evolution of SARS-CoV-2 pneumonia as compared with standard of care (SOC). Considering the high number of current trials testing therapies against COVID-19, we anticipate that we may have to adapt SOC depending on the evolution of guidelines.

## Methods

### Study objectives and endpoints

The principal objective of our trial is to assess the efficacy of 2 units (400–500 mL) of plasma collected in convalescents of COVID-19 infection with a titer greater or equal to 1/320 neutralizing antibodies against SARS-CoV-2 associated with SOC, as compared to SOC alone, to reduce the mortality at day 28 after inclusion of the patients with SARS-CoV-2 pneumonia who require mechanical ventilation.

The secondary objective is to assess the efficacy in terms of clinical outcomes, as assessed by.

1. day-90 mortality
2. duration of mechanical ventilation
3. severity of organ failure
4. use of organ support
5. viral load of the airway
6. inflammatory response
7. hospital length of stay
8. functional, psychological state and quality of life
9. adverse events related to plasma transfusion and to organ support

### Trial design and participants

This is a multicenter two-arm open label randomized superiority trial, with a stratification based on the delay from tracheal intubation to inclusion. The trial will be conducted in the intensive care units (ICUs) of the participating centers in accordance with consensus ethical principles derived from the Declaration of Helsinki, as well as the quality standards of Good Clinical Practice. The participating centers are listed at https://clinicaltrials.gov/ct2/show/NCT04558476. They have been selected according to a feasibility survey, if they had admitted at least 20 patients fulfilling the inclusion criteria during the March–April 2020 period. This trial has been registered at clinicaltrials.gov as NCT04558476. It has been approved by the Central Ethics Committee of the University hospital of Liège, Belgium on September 1, 2020, number # 2020/239. The study is expected to last from September 2020 to September 2022. Each patient will be followed-up for 365 days after enrolment.

### Patient and public involvement

No patient involvement.

### Recruitment and selection of convalescent donors

Convalescent plasma donors will be recruited by the Belgian Red-Cross in a population of patients that were infected with SARS-CoV-2 and recovered, according to national legal requirements to donate blood or plasma. Donors will be adults, asymptomatic with prior diagnosis of COVID-19, at least 28 days will having passed since full recovery. They must not have a history of blood transfusion or tissue or organ transplantation, positive screening for irregular antibodies. Female donors can only be recruited if no history of pregnancy or tested a negative for anti-HLA/HPA/HNA antibodies. Standard donor criteria for blood or plasma donation must be met and informed consent must be obtained in accordance with the Belgian Blood Legislation and national and EU data protection rules.

### Plasma collection from the selected donors

Collection, processing and storage.

The plasma collection will be performed in a dedicated facility, by plasmapheresis, or if apheresis is not sufficient to supply enough plasma, by plasma separation of the whole blood. Eligible plasmapheresis donors are allowed to donate a total volume per year of 650 ml per session, 2 L per month and 15 L per year. The inter-donation interval is at least one week. Plasma obtained by plasmapheresis and processed will be split before freezing into 2–3 separate units (e.g. 3 × 200 ml). Final products will be specifically labelled as COVID-19 Convalescent Plasma and stored in a dedicated location. The processing that is routinely used for pathogen reduction by Methylene Blue will be applied according to standard practice in the blood establishment. The product can be stored for 36 months at a temperature below- 25 °C or for 3 months at a temperature between -18 °C and -25 °C. The date of the positive diagnosis of COVID-19 and the time from diagnosis until resolution of symptoms will be collected from the donors.

### Assessment of the neutralizing antibodies in the donated plasma

A serum sample from all donors will be stored for testing of antibody specificities. The blood service of the Belgian Red-Cross will determine SARS-CoV-2 neutralizing antibody titers to select donors with high titers. The neutralizing antibody titers will be repeated with a maximal interval of one month. Samples of donations done in-between will be stored for later testing. The blood service of the Belgian Red-Cross will qualify donations from donors with neutralizing antibody titers greater or equal to 1/320 as appropriate for this study. In case of shortage of plasma with a titer greater or equal to 1/320 on the moment of patient enrolment, plasma from convalescent donors with a titer of at least 1/80 will be released for transfusion. Virus neutralization test (VNT) is carried out with SARS-CoV-2 strain BetaCov/Belgium/Sart-Tilman/2020/1 in 96-well plates containing confluent Vero E6 cells (ATCC CRL-1586) [12]. We use 8 dilutions of each heat inactivated serum (1:10 to 1:1280—corresponding to final testing dilutions 1:20 to 1:2560), allowing testing 2 samples or controls per plate. In each VNT, a strong, assured positive control serum from Belgian National Reference Centre (Sciensano) is used. Sera are mixed vol/vol with 100 TCID50/reaction of SARS-CoV-2 and incubated 1 h at 37 °C. Then, the serum plus virus mixture is transferred onto the confluent cell monolayer in triplicate. The VNT relies on cytopathic effect (CPE) observation under light microscopy at day 5 pi. Dilutions of serum associated with CPE is considered as negative, while the absence of CPE indicated a complete neutralization of SARS-CoV-2 inoculum (positive). Virus neutralization titer is reported as the highest dilution of serum that neutralized CPE in 50% of the wells. Serum specimens with a titer ≥ 40 are considered to neutralize the virus.

### Study intervention and procedures

The patients hospitalized with a severe COVID-19 infection will be proposed to participate if they fulfill the following criteria:

1. age at least 18 years
2. hospitalization in an intensive care unit participating to the study
3. medical diagnosis with SARS-CoV-2 pneumonia as defined by both:
  - extended interstitial pneumonia on CT scan or a chest X-ray, consistent with viral pneumonia, within 10 days prior to inclusion
  - Positive result of SARS-CoV-2 PCR test, or any emerging and validated diagnostic laboratory test for COVID-19, within 15 days prior to inclusion
4. under mechanical ventilation administered through an endotracheal tube, for less than 5 days
5. Clinical Frailty Scale < 6 before hospitalization [13]
6. informed consent of the patient, or—if impossible—of a relative acting as the legal representative, or—if impossible—of a physician from a non-participating department of the same hospital acting as an impartial witness.
7. absence of pregnancy
8. absence of prior episode of transfusion-related side effect
9. absence of medical decision to limit therapy
10. absence of current participation in another trial testing a COVID-19 therapy

### Participant or legal representative consent, inclusion and randomization process

Prior to request consent from the patient, the investigator will contact the study coordinating center (UCL clinical trial center, Pr Laterre), which will check both the inclusion criteria and the availability of the convalescent plasma of the ABO blood type of the eligible patient on the website of the Belgian Red-Cross. Then, the capability to be informed and to provide their consent will be assessed for all potential patients. If the written consent of the patient cannot be obtained, it will be asked from a relative/legal representative of the patient or from an impartial witness. The witness can be a physician from another department of the hospital. The eligible patients will be unable to speak—due to tracheal intubation—and most often likely not able to understand the information about the benefits and risks of the trial—due to sedation and/or confusion due to COVID-induced sepsis. Their relatives will likely be only contacted by phone and asked to send a written consent by email, according to the guidelines of the participating ICU, which most frequently preclude access of visitors to the hospitals due to the pandemic aspect of COVID-19. In case of inclusion without the written consent of the patient and in case of recovery, his/her consent to continue to participate and/or to use his/her personal data will be requested. The process of randomization will be performed through a standard randomization system (UCL clinical trial center, Pr Laterre) either directly through the eCRF system or by the CCC St Luc. The randomization process will be stratified according to the delay from tracheal intubation to inclusion (“<= 48 h”; “between 48 h and 5 days”). A response to telephone calls for any issue regarding the inclusion criteria will be available 24/7. The treatment (intervention versus control) will be allocated in 2 arms in a 1:1 ratio.

### Study arms

We used blocked randomization to form the allocation list for the two comparison groups. We used a computer random number generator, R software, to select random permuted blocks with a block size of four and an equal allocation ratio. The randomization process was stratified according to the delay from tracheal intubation to inclusion (<= 48 h and between 48 h and 5 days).

When the patient is randomized in the intervention arm, he/she will be administered 2 units (400–500 mL in total) of convalescent plasma. The units of plasma will be shipped by the Belgian Red-Cross to the local blood bank. The treatment will be administered within 24 h of inclusion in the study and within six hour after thawing, through a venous line over at least 2 h. The patients randomized to control arm will not receive convalescent plasma. No cross-over will be allowed between the two arms. The two groups will be treated with the current SOC, made of therapies currently recommended in international guidelines regarding organ failure support and treatment of secondary events and treatment directed against SARS-CoV-2 or the inflammatory response to the infection. At the present time, this treatment includes steroids, in accordance with the recently published “Recovery” trial [14]. In case of evolution of the guidelines due to new scientific release during the study period, the SOC will be adapted in the two treatment groups.

### Evaluation visits

Patients will be evaluated during the screening period for inclusion criteria, on day 1 for the inclusion visit, on days 7, 14 21 28, 90 and 365 for follow-up visits and every day in the ICU to assess the occurrence and duration of organ supportive techniques and secondary events. In case of discharge of the hospital, the visits will be performed either by phone or visual contact. The Table 1 illustrates the schedule of assessments at each phase of the follow-up .

**Table 1:** Schedule of the assessments for each patient during the study

Procedures	Visits								
	Screening	Inclusion	Treatment phase				Follow-up		
		Day 1	Day 7	Day 14	Day 21	Day 28	ICU Discharge	Day 90	Day 365
Informed consent		x							
Eligibility assessment	x	x							
Randomisation		x							
Medical history		x							
Comorbidities									
Prior medications									
APACHE II score		x							
SOFA score		x	x	x	x	x			
WHO disease progression scale		x	x				x		
CRP		x	x	x	x	x			
Viral load (NP or tracheal PCR)									
Clinical frailty scale		x							
Concomitant drugs against SARS-CoV-2	x	x	x	x	x	x			
Biological findings		x^a^	x^b^	x^b^	x ^b^	x^b^			
24 h-diuresis		x	x	x	x	x			
Serum sample for central analysis		x	x			x		x	
Mechanical ventilation		x	x	x	x	x			
Renal replacement therapy		x	x	x	x	x			
Vasoactive drugs		x	x	x	x	x			
ECMO		x	x	x	x	x			
Nosocomial infections		x	x	x	x	x			
Iatrogenic events		x	x	x	x	x			
Adverse events & Serious adverse event		x	x	x	x	x			
ADL scale		x						x	x
Anxiety-depression scale								x	x
EQ-5D-5L scale		x						x	x

^a^Hemoglobin, Hematocrit, Platelet count, White blood cells count, Neutrophil, Lymphocyte, PaO2, FiO2, Na, K, urea, creatinine, protein, bilirubin, ferritin, CRP, D-dimers, fibrinogen, LDH, TGO, TGP, CPK

^b^Creatinine, platelet count, bilirubin, CRP, WBC count, Ferritin, Lymphocyte count

### Data sharing

The data will be shared at the European level in accordance with Belgian and KCE requirements https://ec.europa.eu/health/blood_tissues_organs/covid-19_en. The data shared will be anonymous and include the following ones:

1. Gender, age range (30–39, 40–49 etc.), co-morbidities
2. Transfusion time point (in days from disease onset)
3. Number, volume and anti-body titre of transfused unit(s
4. Therapies administered to the patient in parallel (other than supportive care)
5. Clinical symptoms and laboratory parameters—according to the WHO Ordinal Scale for Clinical Improvement [7] at the following time points:
  - Prior to transfusion
  - 5 days after transfusion
  - At discharge (if the patient survives)
6. Any serious adverse reactions or events possibly linked to the transfusion
7. Length of hospitalisation (if no death).

The de-identified raw data will be available on request to the corresponding author on request at benoit.misset@chuliege.be for the purpose of meta-analyses.

### Data management and monitoring

Measurements will be performed by the investigators and clinical research assistants during their stay in the hospital and during medical visits at each time of endpoints (days 7, 14, 28) in case of hospital discharge, and by phone for the day 90 and for day 365 endpoint. Vital status, dependence on the ventilator and clinical and biological data to calculate the SOFA score will be collected from the patient’s record. Viral load, expressed as cycle threshold, will be assessed from tracheal aspirates or naso-pharyngeal swabs and real time polymerase chain reaction (RT-PCR) assessment by the usual technique of the routine laboratory. Adverse events possibly related to study intervention will be collected prospectively, according to definitions of iatrogenic events, transfusion-related events and nosocomial infections by the investigator and the clinical research assistant in charge of the study. Scales assessing functional state, psychological state and quality of life will be collected over the phone at the day 90 visit and the day 365 visit by a clinical research assistant dedicated to this task by the coordinating center. The adverse events will be collected and severity rating according to Belgian guidelines. Data will be collected from the patients’ records by a clinical research assistant and monitored by the clinical center of UCL team. It will be pseudo-anonymized in a secure database that was built at Saint-Luc Hospital in Brussels, Belgium, and maintained on a dedicated server. The study will be monitored by a DSMB that will include international members, including a statistician, who are not involved in this protocol and independent of the promotor and the funder. The DSMB will review the safety data at least every 6 months and at each interim analysis.

### Adverse events

Patients will be continuously monitored for adverse events. Events related to plasma infusion will be reported to the local transfusion service, which in turn will notify them to the national system for surveillance for hemovigilance. The adverse events due to SOC will be notified in the electronic case record form (eCRF) and reported to the coordinating investigator and to the chair of the DSMB. More details on the protocol can be found in Additional file 1. This protocol complies with the SPIRIT requirements, and the corresponding check-list is provided in Additional file 2.

## Statistical analysis

### Sample size

The primary outcome of the trial is mortality at day 28. Based on prior reports from China, Italy and UK, and consistent with personal observations, we anticipate that the mortality at day 28 will be around 40%. The reduction that we anticipate is empiric and in accordance with the single trial published to date [8]. We consider that a one third relative reduction in 28-day mortality is realistic and clinically relevant. As this reduction might be greater, we plan to perform an interim analysis for ethical reasons. With a two-sided α-risk of 0.05 and a β-risk of 0.20, the minimal number of patients to include to reach significance for a one third relative reduction in 28-day mortality (an absolute reduction of 13.5% day-28 mortality in the intervention group assuming a day-28 mortality of 40% in the control group) is 250 in the control group and 250 in the active group (total number of patients = 500). This number allows for four interim analyses to be performed in addition to the final analysis, respectively after assessment of 100, 200, 300, 400 and 500 patients. Considering that others trials in COVID-19 may result in modification in mortality and therefore in SOC, we anticipate that the number of patients that is necessary to address our hypothesis may vary, and will have to be adapted depending on the results of the interim analyses.

### Statistical analysis plan

Results will be analysed in intention to treat and expressed as means and standard deviations (SD) for quantitative variables with a Normal distribution and as medians and 25th to 75th percentiles for skewed distributed quantitative variables. Qualitative variables will be expressed using counts and percentages.

Mean values between groups will be compared by Student t-test but also by the non-parametric Wilcoxon test when normality assumption is not fulfilled. Proportions will be compared by the chi-square test. Appropriate multivariate analyses will be performed to assess the relationship between outcome and patients’ characteristics and clinical features.

For the primary endpoints, vital status at day 28 in all groups will be calculated with corresponding 95% CI. Comparison between groups will be tested using chi-square test.

Regarding the secondary endpoints, quantitative values between groups will be compared by Student t-test or non-parametric Wilcoxon for the sequential organ failure assessment (SOFA) score (at different time points), viral load of SARS-CoV-2 (at different time points), circulating C-reactive protein (CRP, at different time points), ADL score at day 90, anxiety-depression scale at day 90, sero-conversion. Poisson regression analysis will be considered to analyse number of ventilator-free days up to day 28. Comparison of proportions will be applied to compare location of patient at day 90, as well as presence of various adverse events. If descriptive analysis revealed the presence of potential confounding factors, appropriate multivariate analysis will then be considered.

The two-sided level of significance used in this trial is 5%. Statistical analyses will be carried out using Statistical Analysis System (SAS, version 9.4 for Windows) statistical package.

### Interim analysis plan

Due to ethical considerations of the current situation, we propose to conduct a group-sequential design with several interim analyses. The objective of including interim analyses in the study design is to allow the possibility of stopping the study early if a clinically relevant benefit is already established from a statistical point of view. Conversely, the interim analyses also allow the possibility of an early stopping of the study if the observed benefit, at the time of the interim analysis, is so small as to make it extremely unlikely that the study would succeed, were it continued until full accrual. Finally, the interim analyses will also include a futility stopping rule for the SOC group, which may appear to be unjustified if mortality in that group appears too large to be acceptable.

Specifically, the interim assessment of the SOC group will be based on the numbers of patients and of deaths shown in Table 2. The recommendation will be made to stop accruing patients in the SOC group if the number of deaths observed in this group suggests a true mortality rate in excess of 0.4. The rule for DSMB recommendation shown in Table 2 will not be binding. If the recommendation of the DSMB is to stop the trial, it will be provided to the Ethics Committee by the sponsor. The sponsor shall discontinue—temporarily or definitely—the trial by take into account the recommendations of the DSMB and the Ethics Committee.

**Table 2:** interim analysis and potential recommendation of the DSMB to stop the SOC group

Interim analysis	Number of patients in SOC group	Recommend to stop SOC group if number of deaths	Probability of seeing this number of deaths if true mortality rate ≤ 0.4
1	25	≥ 14	< 0.034
2	50	≥ 26	< 0.031
3	75	≥ 37	< 0.04
4	100	≥ 48	< 0.042

The interim analyses will be performed with 100, 200, 300 and 400 patients evaluable for 28-day mortality. Two Lan-Demets spending functions will be used: one, similar to a Pocock (aggressive) boundary to stop the study for futility, and the other, similar to an O’Brien-Fleming (conservative) boundary to stop the study for extreme efficacy, If the interim analyses take place with increments of exactly 100 patients, and assuming a mortality of 40% in the control group, the recommendations to stop the study for futility or for efficacy are summarized in Table 3. Of note, the trial will reach statistical significance if the absolute difference in 28-day mortality is 9% or larger, which might still be a clinically worthwhile difference. The rules for DSMB recommendations shown in Table 3 will not be binding.

**Table 3:** interim analyses and potential recommendations of the DSMB to stop the trial for futility or efficacy, depending on the observed treatment effect (and associated P-value)

Interim analysis	Number of patients	Recommend stopping for futility if difference in mortality is smaller than	Recommend stopping for efficacy if difference in mortality is greater than
1	100	0.02 (P > 0.83)	-
2	200	0.04 (P > 0.60)	0.22 (P < 0.001)
3	300	0.06 (P > 0.28)	0.14 (P < 0.007)
4	400	0.08 (P > 0.12)	0.11 (P < 0.022)
5 (final)	500	-	0.09 (P < 0.042)

### Provision for adaptive design changes at the time of interim analyses

Because of the considerable uncertainties about the outcomes that will be observed in the two arms of the trial, the DSMB will be allowed to recommend design changes based on the observed data at the times of the interim analyses. These changes may include an adaptation of the sample size or the patient selection. Any such changes are adaptive in nature and will be implemented using appropriate methods to control the impact of these changes on the operating characteristics of the trial (especially the probability of type I error).

### Funding and data sharing with the funder

The trial is funded by the Belgian Health Care Knowledge Center, named KCE, a federal agency which develops the KCE Trials program, consisting in publicly funded, non-commercial, practice-oriented clinical trials. These studies address questions that are generally not studied by industry, despite their high social importance. The University hospital of Liège owns the Study Data, but provides KCE with a royalty-free unrestricted license to use the Study Data for non-commercial public health related purposes.

### Dissemination of results and publication policy

The results will be submitted for presentation to international societies of critical care and/or infectious diseases and for publication to an international scientific medical journal. The authorship will include the members of the writing and/or steering committee, the investigators in the participating centres. The data will be provided to the authors of international collaborative meta-analyses on request.

## Discussion

This study will be the first to assess the efficacy of plasma collected from convalescents of COVID-19 in the population of mechanically ventilated patients. The high mortality of this specific population increases the chance to observe a difference between the two groups. The prospective and randomized design will allow to give a solid answer to the question. A stratification on the delay from mechanical ventilation and inclusion in the study will allow to determine the optimal time to use the convalescent plasma. The participation of 16 centers of all Belgium will insure the external validity of the results and the recruitment of the patients in an acceptable time period. Selecting convalescent plasmas with a high titer of neutralizing antibodies against SARS-CoV-2 will guaranty an homogeneous treatment in the intervention group and prevent the occurrence of the antibody-dependent enhancement of the disease [15]. Lastly, planning iterative interim analyses will allow to stop the trial early in case of an ethical issue induced by the observed results.

## Supplementary information

**Additional file 1**. Detailed protocol.

**Additional file 2**. SPIRIT check-list.

## Data Availability

The data will be shared at the European level in accordance with Belgian and KCE requirements https://ec.europa.eu/health/blood_tissues_organs/covid-19_en. The de-identified raw data will be available on request to the corresponding author on request at benoit.misset@chuliege.be for the purpose of meta-analyses.

## Abbreviations

APACHE II: Acute Physiology And Chronic Health Evaluation II
COVID-19: Coronavirus disease 2019
CPE: Cytopathic effect
CPK: Creatine phosphokinase
CRP: C-reactive protein
DSMB: Data safety monitoring board
ECMO: Extra corporeal membrane oxygenation
eCRF: Electronic case record form
EU: European Union
FiO2: Fraction of inspired oxygen
HLA: Human Leucocyte Antigens
HNA: Human Neutrophil Antigens
HPA: Human Platelet Antigens
ICU: Intensive care unit
KCE: Belgian Healthcare Knowledge Center
LDH: Lactate dehydrogenase
PaO2: Partial pressure of oxygen in arterial blood
RT-PCR: Real time polymerase chain reaction
SARS-CoV-2: Severe acute respiratory syndrome coronavirus 2
SAS: Statistical Analysis System
SGOT: Serum glutamic-oxaloacetic transaminase
SGPT: Serum glutamic pyruvic transaminase
SOC: Standard of care
SOFA: Sequential organ failure assessment
UK: United Kingdom
USA: United States of America
VNT: Virus neutralization test
WBC: White blood cells
WHO: World Health Organisation

## References

[1] Coronavirus COVID-19 (2019-nCoV) [Internet]. [cited 2020 Apr 12]. https://gisanddata.maps.arcgis.com/apps/opsdashboard/index.html#/bda7594740fd40299423467b48e9ecf6

[2] Epistat – Covid-19 [Internet]. [cited 2020 Apr 12]. https://epistat.wiv-isp.be/covid/covid-19.html

[3] ICNARC – Reports [Internet]. [cited 2020 Apr 12]. https://www.icnarc.org/Our-Audit/Audits/Cmp/Reports

[4] Wu C, Chen X, Cai Y, Xia J, Zhou X, Xu S (2020). Risk factors associated with acute respiratory distress syndrome and death in patients with coronavirus disease 2019 pneumonia in Wuhan.

[5] Callaway E (2020). The race for coronavirus vaccines: a graphical guide. Nat Publishing Group.

[6] Shen C, Wang Z, Zhao F, Yang Y, Li J, Yuan J, et al. Treatment of 5 critically ill patients with COVID-19 with convalescent plasma. JAMA. 2020 10.1001/jama.2020.4783 PMC7101507 32219428

[7] Duan K, Liu B, Li C, Zhang H, Yu T, Qu J, et al. Effectiveness of convalescent plasma therapy in severe COVID-19 patients. Proc Natl Acad Sci USA. 2020; 10.1073/pnas.2004168117 PMC7196837 32253318

[8] Li L, Zhang W, Hu Y, Tong X, Zheng S, Yang J (2020). Effect of convalescent plasma therapy on time to clinical improvement in patients with severe and life-threatening COVID-19: a randomized clinical trial. JAMA.

[9] Joyner MJ, Wright RS, Fairweather D, Senefeld JW, Bruno KA, Klassen SA, et al. Early safety indicators of COVID-19 convalescent plasma in 5,000 patients. J Clin Invest. 2020 10.1172/JCI140200 PMC7456238 32525844

[10] Joyner MJ, Senefeld JW, Klassen SA, Mills JR, Johnson PW, Theel ES, et al. Effect of convalescent plasma on mortality among hospitalized patients with COVID-19: initial three-month experience. medRxiv. Cold Spring Harbor Laboratory Press; 2020;2020.08.12.20169359.

[11] Wu F, Liu M, Wang A, Lu L, Wang Q, Gu C (2020). Evaluating the association of clinical characteristics with neutralizing antibody levels in patients who have recovered from mild COVID-19 in Shanghai.

[12] Wu H-S, Chiu S-C, Tseng T-C, Lin S-F, Lin J-H, Hsu Y-H (2004). Serologic and molecular biologic methods for SARS-associated coronavirus infection. Taiwan Emerg Inf Dis.

[13] Guidet B, de Lange DW, Boumendil A, Leaver S, Watson X, Boulanger C (2020). The contribution of frailty, cognition, activity of daily life and comorbidities on outcome in acutely admitted patients over 80 years in European ICUs: the VIP2 study. Intensive Care Med.

[14] RECOVERY Collaborative Group, Horby P, Lim WS, Emberson JR, Mafham M, Bell JL, et al. Dexamethasone in Hospitalized Patients with Covid-19 - Preliminary Report. New Engl J Med. 2020 10.1056/NEJMoa2021436 PMC7383595 32678530

[15] Lee WS, Wheatley AK, Kent SJ, DeKosky BJ (2020). Antibody-dependent enhancement and SARS-CoV-2 vaccines and therapies. Nat Microbiol.

