# Supplementary material for: A multicenter randomized trial to assess the efficacy of CONvalescent plasma therapy in patients with Invasive COVID-19 and acute respiratory failure treated with mechanical ventilation: the CONFIDENT trial protocol
Source: BMC Pulm Med. 2020 Dec 7;20:317. doi: 10.1186/s12890-020-01361-x (PMC7719725; doi:10.1186/s12890-020-01361-x)
Supplement: Supplementary file 1 [file 12890_2020_1361_MOESM1_ESM.pdf]

A multicenter randomized trial to assess the efficacy of CONvalescent plasma therapy in patients with Invasive COVID-19 and acute respiratory failure treated with mechanical ventilation: the CONFIDENT trial.

Acronym / Protocol code	CONFIDENT
Protocol version and date	Version 2.0 – 15/09/2020
Phase	Phase II
Eudract #	2020-003102-31
Name and affiliation of academic promotor	University Hospital of Liège<br>CHU de Liège<br>4000 Liège
Legal representative	Julien Compère, CEO
Coordinating Investigator:	Benoît Misset, MD, PhD<br>Department of Intensive Care<br>CHU de Liège<br>Domaine Universitaire du Sart Tilman, B35<br>4000 Liège<br>Benoit.Misset@chuliege.be

eCRF, Randomization, Monitoring, Data management	Clinical Coordinating Center (CCC)<br>Saint Luc Clinical Coordinating Center<br>Avenue Hippocrate 10<br>1200 Brussels, Belgium<br>Director Pierre-François Laterre<br>Tel: +32 (2) 764 27 80<br>Fax: +32 (2) 764 27 57<br>Email: succc@uclouvain.be
Study Statistician	Anne-Françoise Donneau, MD, PhD<br>Liège University
Co-investigators:	Multicentric trial in Belgium
Financial/Material Support:	KCE contacts<br>France Vrijens, Head of the KCE trials program<br>Nelle Stocquart, researcher, KCE trials program<br>Hilde Nevens, KCE trials program

1. Title Page

Number of participating centers : 16

Steering Committee

Intensivists

Benoît Misset (Liège)

David Grimaldi (Erasme, Bruxelles)

Eric Hoste (Ghent)

Pierre François Laterre (St Luc, Bruxelles)

Geert Meyfroidt (Leuven)

Michel Moutschen, MD, PhD (Infectious diseases, Liège)

Tome Najdivski (Belgian Red-Cross)

Veerle Compernolle (Belgian Red-Cross)

André Gothot (Transfusion, Liège)

Anne-Françoise Donneau (Biostatistics, Liège)

Scientific advisors Jean-Louis Vincent, Michel Goldman

Data Safety Monitoring Board

Jean Chastre (Intensive care, Paris)

Tom Van der Poll (Infectious diseases, Amsterdam)

Jérôme Pugin (Intensive care, Geneva)

Olivier Lesieur (Intensive care, La Rochelle, Ethic Committee of the SRLF)

Murielle Mauer (biostatistics, EORTC)

2. Signature pages

A multicenter randomized trial to assess the efficacy of CONvalescent plasma therapy in patients with Invasive COVID-19 and acute respiratory failure treated with mechanical ventilation: the CONFIDENT trial.

Protocol Coordinating Investigator signature page

I agree to personally conduct or supervise this study and to ensure that all co-investigators, and study staff assisting in the conduct of this study are informed about their obligations in meeting their commitments.

I will conduct the study in accordance with Good Clinical Practice, the Declaration of Helsinki, and the moral, ethical and scientific principles that justify medical research. The study will be conducted in accordance with all relevant laws and regulations relating to clinical studies and the protection of patients.

I will ensure that the requirements relating to Ethics Committee review and approval are met.
I agree to maintain adequate and accurate records and to make those records available for audit and inspection in accordance with relevant regulatory requirements.

I agree to promptly report to the EC any changes in the research activity and all unanticipated problems involving risks to human subjects or others. Additionally, I will not make any changes in the research without EC approval, except where necessary to ensure the safety of study participants.

Investigator:

Name: Prof. Dr. Benoît Misset
Function: Intensive Care Doctor
Institution: CHU de Liège

Name	Signature	Date

A multicenter randomized trial to assess the efficacy of CONvalescent plasma therapy in patients with Invasive COVID-19 and acute respiratory failure treated with mechanical ventilation: the CONFIDENT trial.

Protocol Site Principal Investigator signature page

I certify that I will conduct the study in compliance with the protocol, any amendments, GCP and the declaration of Helsinki, and all applicable regulatory requirements.

Investigator:

Name:

Function:

Institution:

Name	Signature	Date

3. Protocol synopsis

Name of Academic promotor/Coordinator/ Sponsor: CHU de Liège / Pr. Benoit Misset
Name of test drug/investigational product/device: convalescent plasma from COVID-19
Name of Active Ingredient: human antibodies against SARS-CoV-2
Title of Study: A multicenter randomized trial to assess the efficacy of CONvalescent plasma therapy in patients with Invasive COVID-19 and acute respiratory failure due to treated with mechanical ventilation: the CONFIDENT trial.
Indication: Plasma therapy for mechanically ventilated COVID-19 patients
Number of study centers : 16
Publication (reference)

Studied period:<br>(date of first enrolment): September 1,2020<br>(date of last patient-last visit (LPLV)): August 1, 2022	Phase II study

Objectives:

- Primary: The principal objective of the CONFIDENTtrial is to assess the efficacy of two units (400-500 mL in total) of convalescent plasma, as compared to Standard of Care (SoC), to reduce day-28 mortality in patients with SARS-CoV-2 pneumonia who require mechanical ventilation.

- Secondary:

- the efficacy of convalescent plasma
    - to reduce day-90 mortality
    - to reduce the duration of mechanical ventilation over the 28 days after inclusion,
    - to reduce the severity of organ failure over the 28 days after inclusion,
    - to reduce the use of organ support over the 28 days after inclusion
    - to reduce the viral load, expressed as cycle threshold, over the 28 days after inclusion
    - to reduce the intensity of the inflammatory response during the 28 days following inclusion.
    - to reduce the acute care hospital length of stay
    - to increase the functional, psychological state and quality of life at day 90 after inclusion
    - to assess the impact of convalescent plasma on sero-conversion against SARS-CoV-2
- the incidence of adverse events related to blood transfusion
- the incidence of adverse events related to organ support

Hypothesis: Passive immunization with plasma collected from patients having contracted COVID-19 and developed specific antibodies may alleviate symptoms and viral load of SARS-CoV-2 and reduce mortality

in patients treated with mechanical ventilation for severe respiratory failure during the evolution of SARS-CoV-2 pneumonia.
Study Design (Treatment Schema): Randomized open label trial
Number of patients (planned): 500
Endpoints :<br>- Primary:<br>- vital status (dead/alive) at day 28<br>- Secondary:<br>- day-90 mortality<br>- number of ventilator-free days at day 28<br>- number of renal replacement therapy free days at day 28<br>- number of vasopressors free-days at day 28<br>- use of ECMO before day 28<br>- value of the SOFA score at days 7, 14 and 28<br>- changes in SOFA scores (delta SOFA) over 7, 14 and 28 days<br>- assessment of the SARS-CoV-2 viral load, expressed as cycle threshold, [2] in the tracheal aspirates (for intubated patients) or nasopharyngeal swabs (for extubated patients) at days 7, 14 and 28<br>- blood C reactive protein (CRP) and ferritin concentrations, and lymphocyte count at days 7, 14 and 28<br>- length of stay in the acute care hospital<br>- location of the patient at day 90: acute care hospital, post-acute care hospital, long-term residency, home<br>- activity of daily living (ADL) functional score at day 90 and at day 365<br>- anxiety-depression evaluation scale at day 90 and at day 365<br>- quality of life scale at day 90 and at day 365<br>- transfusion-related adverse events within 28 days<br>- Sero-positivity to SARS-CoV-2 at days 1, 7, 28 and 90
Diagnosis: severe SARS-CoV-2 pneumonia
Main criteria for inclusion:<br>- aged at least 18 years<br>- hospitalized in an intensive care unit participating to the study<br>- diagnosed with SARS-CoV-2 pneumonia as defined by both:<br>o extended interstitial pneumonia on CT scan or chest X-ray, consistent with viral pneumonia, within 10 days prior to inclusion

o positive result of SARS-CoV-2 PCR test, or any emerging and validated diagnostic laboratory test for COVID-19 within 15 days prior to inclusion<br>- under mechanical ventilation administered through an endotracheal tube, for no more than 5 days<br>- Clinical frailty scale < 6<br>- Written consent of the patient, or – if impossible - of a relative acting as the legal representative, or – if impossible - of a physician from a non-participating department of the same hospital acting as an impartial witness (see trial procedures).
IP (Investigational Product) dosage and mode of administration: 2 units (400-500 mL in total) of convalescent plasma collected from 2 different donors preferably having recovered from COVID-19 with a titer greater or equal to 1/320 preferentially. In case of shortage of plasma with a titer greater or equal to 1/320 on the moment of patient enrolment, plasma from convalescent donors with a titer of at least 1/80 will be released for transfusion. The plasma will be ABO compatible. The treatment will be administered within 24 hours of inclusion in the study, through a venous line over at least 2 hours. Comparators = standard of care
Procedures: Visits at days 7, 21, 14, 28,90 and 365.
Duration of treatment = 2 hours, duration of surveillance per patient = 365 days
Statistical Considerations: Intention to treat analysis, expected mortality at day 28 = 40%, expected relative reduction with study intervention = 30 %, α = 5%, β = 20%, number of subject to include = 500. Interim analyses at 100, 200, 300 and 400 patients included.

4. Table of contents

1. TITLE PAGE 3

2. SIGNATURE PAGES 4

3. PROTOCOL SYNOPSIS 6

4. TABLE OF CONTENTS 9

5. STUDY GLOSSARY 11

6. ETHICAL AND REGULATORY CONSIDERATIONS 12

7. OBJECTIVES 13

7.1. PRIMARY 13
7.2. SECONDARY 13
7.3. ENDPOINTS 13

8. BACKGROUND INFORMATION AND SCIENTIFIC RATIONALE 15

9. INVESTIGATIONAL PLAN 19

9.1. DESIGN 19
9.2. DESCRIPTION OF POPULATION 19
9.3. PARTICIPANTS ELIGIBILITY 19
9.3.1 Inclusion criteria 19

9.3.2 Exclusion criteria 19

9.4. TREATMENTS 20
9.4.1 Treatment Administered 20

9.4.2 Identity of Investigational Product (s) 22

9.4.3 Selection of Doses in the Study 22

9.4.4 No blinding 22

9.4.5 Prior and concomitant therapy 22

9.4.6 Treatment Compliance 22

9.5. STUDY PROCEDURES 22
9.5.1 Availability of the study product 22

9.5.2 Participant consent 23

9.5.3 Process of randomization 23

9.5.4 Visits 23

9.5.5 Data to be shared at the European level regarding convalescent plasma use 31

9.5.12 Assessment visits in case of discharge before day 90 32

9.5.13 Laboratory tests 32

9.5.14 Premature discontinuation of trial treatment 32

9.6. EFFICACY AND SAFETY VARIABLES 35
9.6.1 Efficacy and Safety Measurements Assessed and Flow chart 35
9.6.2 Appropriateness of Measurements 35
9.6.3 Primary Efficacy Variable 35
9.7. SAFETY REPORTING 35
9.7.1 Definitions 36
9.7.2 Safety reporting procedures 36
9.8. CONFIDENTIALITY AND DATA HANDLING 37
9.9. STATISTICAL ANALYSIS 37
9.10. CHANGES IN THE CONDUCT OF THE STUDY OR PLANNED ANALYSES 39
9.11. PROTOCOL AMENDEMENTS 39
10. STUDY PATIENTS 40
10.1. DISPOSITION OF PATIENT 40
10.2. PROTOCOL DEVIATIONS 40
10.3. DATA MANAGEMENT RESPONSIBILITIES 40
10.4. STUDY DATA, DATA OWNERSHIP AND DATA SHARING WITH KCE 40
11. FINANCE AND INSURANCE 42
12. DISSEMINATION OF RESULTS AND PUBLICATION POLICY 43
13. ARCHIVING 44
14. STUDY REPORT 45
15. LITERATURE REFERENCES 46
16. APPENDIX 49

5. Study Glossary

Abbreviation	Meaning
ADE	Antibody Dependent Enhancement
ADL	Activity of Daily Living
AE	Adverse Event
ARDS	Acute Respiratory Distress Syndrome
COVID-19	Coronavirus Disease 2019
CRF	Case Report Form
CRP	C-Reactive Protein
EC	Ethics Committee
ECMO	ExtraCorporel Membrane Oxygenation
FAMHP	Federal Agency for Medicines and Health Products
GCP	Good Clinical Practice
HFO	High Flow Oxygen
KCE	Belgian Healthcare Knowledge Centre
ICH	International Conference on Harmonization
ICU	Intensive Care Unit
IgG	Immunoglobulin type G
IVIG	Intravenous Immunoglobulin Therapy
MERS	Middle East Respiratory Syndrome
NIV	Non Invasive Ventilation
PRNT50	Plaque Reduction Neutralization Test 50 %
RT-PCR	Reverse Transcription Polymerase Chain Reaction
SAE	Serious Adverse Event
SARS	Severe Acute Respiratory Syndrome
SoC	Standard of Care
SARS-CoV-2	SARS-coronavirus-2
SOFA	Sequential Organ Failure Assessment
TRALI	Transfusion-Related Acute Lung Injury
UK-ICNARC	United-Kingdom Intensive Care National Audit & Research

6. Ethical and regulatory considerations

- The Principal Investigator at each center will ensure that this study is conducted in agreement with the Declaration of Helsinki as well as the laws and regulations of the country, whichever provides the greatest protection for the patient.

- All study documents (protocol, any protocol amendment, informed consent form and other relevant documents (eg. recruitment advertisements…) will be submitted to the Ethics Committee for formal approval to conduct the study. The decision of the EC concerning the conduct of the study will be made in writing to the promotor. All correspondence with the IRB/IEC should be retained in the Trial Master File.

- The protocol will be approved by the local Ethics Committee, with the Comité d'Ethique Hospitalo-Facultaire de Liège serving as the central Ethics Committee.

- The protocol will be registered in ClinicalTrial.gov before initiation.

- The study will be conducted in accordance with legal and regulatory requirements (Belgian law of 7 May 2004, Patient rights (August 2002), Private life (RD 2001), HBM (Human Body Material, law of 19 December 2008), RGPD (25 May 2018), as well as the Guidelines for Good Clinical Practice (International Conference on Harmonization), and the last version of Declaration of Helsinki (World Medical Association).

7. Objectives

7.1. Primary

The principal objective of the CONFIDENT trial is to assess the efficacy of 2 units (400-500mL) of plasma collected in convalescents of COVID-19 infection with a titer greater or equal to 1/320 (plaque reduction neutralization test 50) neutralizing antibodies against SARS-CoV-2 with the standard of care, as compared to standard of care, to reduce the mortality at day 28 after inclusion of the patients with SARS-CoV-2 pneumonia who require mechanical ventilation. In case of shortage of plasma with a titer greater or equal to 1/320 on the moment of patient enrolment, plasma from convalescent donors with a titer of at least 1/80 will be released for transfusion. The standard of care will be made of therapies currently recommended in international guidelines regarding organ failure support and treatment of secondary events. At the present time, no treatment is directed against SARS-CoV-2 or the inflammatory response characteristic of this specific disease. In case of evolution of the guidelines due to new scientific release during the study period, the standard of care will be adapted in the two treatment groups.

7.2. Secondary

The secondary objectives are to assess:

- the efficacy of convalescent plasma
    - to reduce day-90 mortality
    - to reduce the duration of mechanical ventilation over the 28 days after inclusion,
    - to reduce the severity of organ failure over the 28 days after inclusion,
    - to reduce the use of organ support over the 28 days after inclusion
    - to reduce the viral load, expressed as cycle threshold, over the 28 days after inclusion
    - to reduce the intensity of the inflammatory response during the 28 days following inclusion.
    - to reduce the acute care hospital length of stay
    - to increase the functional, psychological state and quality of life at day 90 and at day 365 after inclusion
    - to assess the impact of convalescent plasma on sero-conversion against SARS-CoV-2
- the incidence of adverse events related to plasma transfusion
- the incidence of adverse events related to organ support.

7.3. Endpoints

The <u>principal endpoint</u> will be vital status (dead/alive) at day 28

The <u>secondary endpoints</u> will be

- day-90 mortality
- number of ventilator-free days at day 28
- number of renal replacement therapy-free days at day 28
- number of vasopressors-free days at day 28
- use of ECMO before day 28
- value of the SOFA score at days 7, 14 and 28 [1] (see appendix A).
- changes in SOFA scores over the following 7, 14 and 28 days
- value of the SARS-CoV-2 viral load, expressed as cycle threshold, [2] in the tracheal aspirates (for intubated patients) or nasopharyngeal swabs (for extubated patients) at days 7, 14 and 28
- blood C reactive protein (CRP) concentrations at days 7, 14 and 28
- length of stay in the acute care hospital
- location of the patients at day 90: acute care hospital, post-acute care hospital, long-term residency, home
- WHO progression scale prior transfusion, at day 7 and at discharge (see appendix B)
- Hospital Activity of Daily Living (ADL) functional score at day 90 and at day 365. [3] (see appendix C).
- Anxiety-depression evaluation scale at day 90 and at day 365. [4] (see appendix D).
- Quality of life scale (EQ-5D-5L) at day 90 and at day 365. [5] (see appendix E).
- Transfusion-related adverse events within 7 days
- Sero-positivity to SARS-CoV-2 at days 1, 7, 28 and 90
- Adverse events related to standard of care (mechanical ventilation, prolonged decubitus, prone positioning…) such as:
    - Ventilator associated pneumonia
    - Secondary bacteremia [6]
    - Deep vein thrombosis and pulmonary embolism
    - Pressure ulcers, stage 2 to 4 (*i.e.* loss of skin, of tissue or of bone) [7]
    - Ventilator induced baro-traumatism (pneumothorax, pneumo-mediastinum, sub-cutaneous emphysema) [8]
    - Accidental ablation of tracheal or vascular device [9]
    - Ventilator induced baro-traumatism (pneumothorax, pneumo-mediastinum, sub-cutaneous emphysema) [8]
    - Accidental ablation of tracheal or vascular device [9]

8. Background Information and Scientific Rationale

The COVID-19 pandemic started in China in December 2019 and spread to European countries in early 2020, affecting most severely Italy, Spain and France. Belgium was affected during the same wave and first deaths due to COVID-19 were reported in early March 2020. As of September 10, 2020, almost 28 million cases, including 900,000 deaths, have been confirmed over the world and almost 90,000 cases, including 9,900 deaths, have been declared in Belgium [10].

Current data from the Belgian surveillance system indicate that 20% of the hospitalized patients are in ICUs [11]. Hospital deaths occur either in the ICU (13 / 31, 31 % in Liège University Hospital, personal data) or in non-ICU wards for those patients who would not benefit from intensive care as anticipated by the medical team [12]. Based on United Kingdom ICNARC reports, and according to personal observations in Belgium, the COVID-19 ICU patients require mechanical ventilation in 60% of cases, particularly when they are referred directly by the emergency medical service. According to different series, forty to sixty percent of the mechanically ventilated patients are likely to die, most of them when mechanical ventilation is instituted early (30% survival rate) rather than late in the course of the disease (70 % survival rate) [13]. Similar observations have been made in China [14]. Besides the direct cost in human lives, the current situation in European countries has led to a halt in most of medical and scheduled surgical practices for non COVID-19 patients and to a doubling of the number of ICU beds. The nursing and medical manpower per patient in ICUs has also been doubled due to the intensity of care that patients require with the use of invasive ventilation and the practice of alternate prone positioning for severe ARDS [15].

Several epidemiologic projections suggest that, in the absence of an effective antiviral vaccine or of therapeutic agents, the human cost may reach as many as 45 million deaths until the global population has developed self-immunization. Preliminary data suggest that immunization occurs within 15 to 20 days of illness in most patients [16] but data in the community are still lacking. The potential duration of the current pandemic is largely unknown and epidemics with coronaviruses are likely to recur. A race for developing active therapies and vaccines started since the onset of the pandemic [16]. The potential therapeutic agents are directed against either the virus itself or the mediators of the exacerbated host response observed in the most severe cases [17]. Current proposed treatments are under evaluation and new therapies and vaccines directed against SARS-CoV-2 are at preliminary steps of development [18]. Therapy with plasma collected from convalescent patients has been proposed to provide passive immunization to the patients at risk of developing severe COVD-19 in small pilot uncontrolled studies [19,20].

Convalescent plasma therapy also called immune plasma therapy has been used to prevent or treat numerous viral diseases for over a century. Besides historical records in rabies, polio, measles, hepatitis or influenza, convalescent plasma therapy has been used successfully against Ebola and during the two previous coronavirus epidemics: SARS-CoV in 2002 and MERS. In a retrospective study, Cheng et al. report that amongst the 339 patients admitted for SARS at the Prince of Wales hospital in Hong-Kong, 80 received convalescent plasma. Mortality in this group was 12.5% and considered lower than the mortality rate of SARS in Hong-Kong (17%) [21]. Interestingly, patients with a good outcome received plasma at day 11.7±2.3 vs 16.0±6.0 in the other ones. The volume administered was 250 to 300 mL and no adverse effects were observed. A meta-analysis published in 2015 on 27 publications describing the effect of convalescent plasma in severe acute respiratory infections of viral origin (including SARS-CoV, H1N1 and H5N1 influenza) similarly reported an overall reduction of mortality (OR 0.25 (0.14-0.45) and no adverse effect [22].

In COVID-19, Shen et al. recently reported 5 patients under mechanical ventilation who received 400 ml convalescent plasma between 10 and 22 days after admission. SOFA scores were 5, 10, 3, 3 and 2, respectively. Bacterial infection was documented in two of them. All of them were treated with lopinavir/ritonavir and IFN-β1. ARDS resolved in four patients and 3 of them could be weaned from mechanical ventilation at the time of publication [19]. Duan et al. reported 10 cases of patients with severe infection receiving convalescent plasma between 11 and 20 days after the onset of symptoms. Prior to treatment, three patients received mechanical ventilation, three received high-flow nasal cannula oxygenation, and two received conventional low-flow nasal cannula oxygenation. Three patients were discharged and the seven others improved and were ready to discharge at the time of publication [20].

In all the published cases of COVID-19 patients, convalescent plasma was associated with a reduction of viral load despite all the patients were receiving various antivirals (including lopinavir/ritonavir or remdesivir). In the study by Shen et al, the Ct value increased in all patients at day 1 post transfusion up to 40 in all patients at day 12 [19]. Duan et al. observed that 10/10 patients reached undetectability 2 to 6 days after the administration of convalescent plasma [20]. A direct antiviral effect of neutralizing antibodies within the convalescent plasma is therefore likely to play a central role in the beneficial effect of the treatment. Nevertheless, it cannot be ruled out that other mechanisms could participate in the effect. It is well known that IV immunoglobulins have a potent anti-inflammatory effect by various mechanisms including engagement of inhibitory FcγR on macrophages and regulatory T cells [23]. Although the amount of IgG present in two units of plasma is clearly lower than the dose of IgG administered during IVIG therapy, such an effect could take place during treatment with convalescent plasma. Other mechanisms could also intervene. Abnormalities of coagulation and complement activation have been described in Covid [24] and are clearly amenable to improvement after plasma administration. In addition, inhibition of ACE2 enzymatic activity by viral infection has been proposed as a central mechanism in the pathogenesis of COVID-19 [25]. Since ACE2 catalytic activity can be

demonstrated in the plasma due to shedding of the ectoenzyme [26], a beneficial antibody-independent effect of plasma therapy cannot be excluded.

Besides rare adverse effects associated with plasma therapy in general, including transfusion- related acute lung injury (TRALI) [27], convalescent plasma therapy raises the issue of potential *antibody dependent enhancement* (ADE). ADE is the paradoxical exacerbation of an infectious disease due to the appearance of specific antibodies produced by the infected subject. It has been observed in several viral diseases including Dengue, Ebola, HIV, Sars CoV and MERS CoV. In coronavirus infections, the main mechanism involves a facilitation of virus entry in FcγR expressing cells by antibodies directed against the spike protein but with a suboptimal neutralizing affinity [28]. Observations in dengue also suggest that sub-neutralizing antiviral antibodies could also contribute to inhibit IFN-γ secretion and cytotoxic T cell differentiation [29]. Although ADE clearly plays an important role in the pathogenesis of viral infections, it is important to state that it has never been observed after convalescent plasma therapy.

In the Shen's study [19], the donors had been asymptomatic for at least 10 days and had a neutralizing antibody titer higher than 1/40. No precision is given regarding the severity of their symptoms. In the study by Duan et al [20], 40 donors were included. The donor's blood was collected after 3 weeks of illness and 4 days of hospital discharge. 39 donors had a neutralizing antibody titer higher than 1/160.

In a most recent survey, Joyner observed 2 serious adverse events directly related to convalescent plasma administration for COVID-19 in a cohort of 5,000 patients (0.04 %) treated in the USA [30]. In a series of 175 patients with mild COVID-19 from Shangaï, China, Wu et al [31] observed that the titers of neutralizing antibodies assessed at hospital discharge to SARS-CoV-2 varied substantially. In 11 patients with sequential assessments, high titers over 1/256 were measured as early as 10 to 16 days of disease onset.

Hypothesis

Our hypothesis is that passive immunization with plasma collected from patients having contracted COVID-19 and developed specific antibodies may alleviate symptoms and viral load of SARS-CoV-2 and reduce mortality in patients treated with mechanical ventilation for severe respiratory failure during the evolution of SARS-CoV-2 pneumonia as compared with standard of care. Considering the high number of current trials testing therapies against COVID-19, we anticipate that we may have to adapt the standard of care depending on the evolution of guidelines.

Current trials testing convalescent plasma therapy in COVID-19 patients

As of April 25, 2020, 41 randomized trials testing the use of convalescent plasma in COVID-19 patients have been declared on Clinicaltrials.gov. Four of these include critically ill patients. NCT 04362176 (Vanderbilt, Nashville, TN, USA) plans to include 500 patients and compare (1:1) convalescent plasma

to placebo (Ringer Lactate), NCT04359810 (Columbia, New-York City, NY, USA) plans to include 105 patients and compare (2:1) 1 unit of convalescent plasma to 1 unit of fresh frozen plasma. NCT 04346446 (New Dehli, India) is open label and plans to include 20 patients. NCT04381858 (Mexico) plans to include 500 patients and compare convalescent plasma vs human immunoglobulin. As of August 28, 63 RCT have been posted on Clinicaltrials.gov. Eighteen trials plan to enroll at least 300 patients. None of them addresses specifically mechanical ventilated patients.

9. Investigational plan

9.1. Design

- Inclusion and randomization

After fulfilling the inclusion criteria, patients will be included by the local investigator in each participating center and randomized through a standard randomization system (UCL clinical trial center, Pr Laterre) into 2 arms (1:1) to receive 2 units (400-500 mL in total) of convalescent plasma plus standard of care from 2 different donors preferably (intervention group) or control treatment made of standard of care alone.

9.2. Description of population

- The study population will be patients with SARS-CoV-2 pneumonia associated with acute respiratory failure and requiring mechanical ventilation
- Number of patients planned: 500 (1 intervention group of 250; 1 control group of 250)

9.3. Participants eligibility

9.3.1 Inclusion criteria

We will include patients with the following criteria:

- age at least 18 years
- hospitalization in an intensive care unit participating to the study
- medical diagnosis with SARS-CoV-2 pneumonia as defined by both:
    - extended interstitial pneumonia on CT scan or a chest X-ray, consistent with viral pneumonia, within 10 days prior to inclusion
    - Positive result of SARS-CoV-2 PCR test, or any emerging and validated diagnostic laboratory test for COVID-19, within 15 days prior to inclusion
- under mechanical ventilation administered through an endotracheal tube, for no more than 5 days
- prior Clinical Frailty Scale < 6 [32] (see appendix F).
- written consent of the patient, or – if impossible - of a relative acting as the legal representative, or – if impossible - of a physician from a non-participating department of the same hospital acting as an impartial witness (see trial procedures).

9.3.2 Exclusion criteria

- Pregnancy
- Prior episode of transfusion-related side effect
- Medical decision to limit therapy

- Current participation in another trial testing a COVID-19 therapy

9.4. Treatments

9.4.1 Treatment Administered

Collection of convalescent plasma

Donor eligibility

Convalescent plasma donors will be recruited in a population of patients that were infected with SARS-CoV-2 and recovered. Potential donors must at least fulfill national legal requirements for eligibility of donors to donate blood or plasma. The collection of blood products is defined by the Belgian Blood legislation (Law July 5th 1994, and R.D. April 4th 1996).

Potential donors can be identified through collaboration with treating hospitals or practitioners or via direct search for patients cured from COVID-19.

Personal data sharing strategies must comply with national and EU data protection rules.

The following criteria for donor eligibility will be applied additional to national legal requirements:

1. a asymptomatic donor with prior diagnosis of COVID-19 documented by a laboratory test or a clinical picture with radiological confirmation of COVID-19.
2. at least 28 days will have passed since full recovery and disappearance of the symptoms. Blood establishments might modify the timing of plasma collection when findings regarding the timing of optimal and maximal antibody production in those who have recovered from COVID-19 become available and is confirmed acceptable by AFMPS/FAGG.
3. Donors without a history of blood transfusion or without history of tissue or organ transplantation.
4. Donors without positive screening for irregular antibodies.
5. Female donors can only be recruited if either no history of pregnancy or if tested and found negative for anti-HLA/HPA/HNA antibodies using a validated assay. Standard donor criteria for blood or plasma donation must be met.
6. Informed consent in accordance with the Belgian Blood Legislation and national and EU data protection rules.

Plasma collection:

Collection, processing and storage

Donors will ideally donate plasma by plasmapheresis, but where apheresis is not sufficient to supply enough plasma, whole blood can also be collected, with plasma separation in the blood establishment. Eligible plasmapheresis donors are allowed, according to Belgian Blood legislation, to donate a total volume of 650 ml per session, 2 liters per month and 15 liters per year. The inter-donation interval is at least one week. Plasma obtained by plasmapheresis and processed will be split before freezing into 2-

3 separate units (e.g. 3x200 ml). Final products will be specifically labelled as COVID-19 Convalescent Plasma and stored in a dedicated location. The processing that is routinely used for pathogen reduction by Methylene Blue will be applied according to standard practice in the blood establishment.
The production process and product characteristics will comply with current Belgian Blood legislation. For this trial, volumes will be between 200 and 250 ml. The product can be stored for 36 months at a temperature below- 25°C or for 3 months at a temperature between -18°C and -25°C.
Data collected from donors
The following data will be collected:

- Date of nasopharyngeal swab that subsequently proved SARS-CoV-2-positive by PCR (or alternative date of radiological confirmation)
- Time from diagnosis until resolution of symptoms

Testing of donated plasma
Defined SARS-CoV-2 neutralizing antibody titers will be measured in the donated plasma. A Blood or Plasma sample from all donors will be stored in tube with gel for that purpose and for testing of antibody specificities.
Blood establishments will determine SARS-CoV-2 neutralizing antibody titers at or prior to the first donation to allow the selection of donors with high titers. The neutralizing antibody titers will be repeated with a maximal interval of one month. Samples of donations done in-between will be stored for later testing.
The Blood Establishment will qualify donations from donors with neutralizing antibody titers greater or equal to 1/320 as appropriate for this study. In case of shortage of plasma with a titer greater or equal to 1/320 on the moment of patient enrolment, plasma from convalescent donors with a titer of at least 1/80 will be released for transfusion.

If an adequate correlation between neutralizing activity and Elisa antibody testing for SARS-CoV2 were to be demonstrated by a validation report to be approved by the sponsor, this assay could replace the test for neutralizing antibodies. Additional archive samples of the donated plasma will be saved for reference studies e.g. frozen aliquots from plasma samples taken at the time of donation.
When the measured neutralizing activity in the collected plasma is considered too low, the plasma will be made available for other use.

Convalescent Plasma and Dosing Regimen
Patients in the intervention arm will get 2 units (400-500 mL in total) of convalescent plasma. A single unit has a volume that ranges from 200mL to 250mL. Determination of ABO compatibility will follow local procedures and plasma will be transfused within six hour after thawing. Plasma will be provided by the

local blood bank. Convalescent plasma will be stored in a limited number of places. Products can only be shipped to the local site only when the patient is randomized.

9.4.2 Identity of Investigational Product (s)

Intervention arm: 2 units (400-500 mL in total) of convalescent plasma. The treatment will be administered within 24 hours of inclusion in the study, through a venous line over at least 2 hours. (to prevent potential adverse effect of fluid overload).

Control arm: no plasma

9.4.3 Selection of Doses in the Study

Interventional group: 2 units (400-500 mL in total) of convalescent plasma with at least 1/320 $PRNT_{50}$ of neutralizing antibodies. Each unit will preferably come from two different donors. Control group: no plasma.

9.4.4 No blinding

We chose not to blind the treatments because the principal endpoint (day 28 mortality) is an objective one.

9.4.5 Prior and concomitant therapy

At the present time, no specific treatment has been proven to improve the outcome of COVID-19 critically ill patients beside the conventional support of organ failure. The treatments administered in compassionate use on a frequent basis (*i.e.* hydroxychloroquine or azithromycin) or on selected cases (*i.e.* anti-HIV or Ebola antivirals, anti-cytokines or anti-cytokine receptors …) are likely to be dispatched similarly in the randomization arms and can be used in the patients included in this trial. Their use will be assessed and provided in the descriptive results of the population.

No particular drug-drug interactions are anticipated.

9.4.6 Treatment Compliance

The patients included will be totally dependent from the organ supply they will receive for life support in the intensive care unit. The treatment will be administered in a single administration. The issue of compliance is therefore not applicable.

9.5. Study Procedures

9.5.1 Availability of the study product

Prior to request informed consent from the patient, his/her representative or a impartial witness (see below), the investigator will contact the CCC St-Luc. The CCC St Luc will check the eligibility of the patient with the local investigator or his/her representative for the study. The CCC St Luc will check the

website of the Red-Cross to ascertain that the convalescent plasma of the patient's ABO group at a titer of 1/320 is available for delivery to the site. In case of shortage of plasma with a titer greater or equal to 1/320 on the moment of patient enrolment, plasma from convalescent donors with a titer of at least 1/80 will be released for transfusion. If the patient is eligible and the plasma is available, the informed consent will be obtained according to the procedure indicated in the "participant consent" paragraph.

9.5.2 Participant consent

The eligible patients will be unable to speak – due to tracheal intubation - and most often likely not able to understand the information about the benefits and risks of the trial – due to sedation and/or confusion due to COVID-induced sepsis. Their relatives will likely be only contacted by phone according to the guidelines of the participating ICU, which most frequently preclude access of visitors to the hospitals due to the pandemic aspect of COVID-19.

The capability to be informed and to provide their consent will be assessed for all potential patients. If the written consent of the patient cannot be obtained, it will be asked from a relative / legal representative of the patient over the phone (preferably through video conference) and the form will be sent for signature to the relative and returned by the him/her by email. In the absence of written consent due to incapacity of the patient or to impossibility to have a contact with the relative / legal representative, the investigator will have to obtain the written approval from an impartial witness. The witness can be a physician from another department of the hospital.

In case of inclusion without the written consent of the patient and in case of recovery, his/her consent to continue to participate and/or to use his/her personal data will be requested.

9.5.3 Process of randomization

The process of randomization will be performed through a standard randomization system (UCL clinical trial center, Pr Laterre) either directly through the eCRF system or by the CCC St Luc. The randomization process will be stratified according to the delay from tracheal intubation to inclusion ("<= 48 hours"; "between 48 hours and 5 days"). A response to telephone calls for any issue regarding the inclusion criteria will be available 24/7 (see "availability of the study product" paragraph).

The treatment (intervention versus control) will be allocated in 2 arms in a 1:1 ratio. The randomization table will be made of permutated block size provided by the statistician.

9.5.4 Visits

9.5.4.1 Screening

The principal investigator of each participating center will organize the detection of potentially eligible patients, based on medical history and inclusion criteria available in the patient's record, gather consent to participate according to the study protocol. The response must be yes to all the following questions:

- Age > 18 years
- Hospitalisation in a ICU participating to the study
- extended interstitial pneumonia on CT scan or chest X-ray, consistent with viral pneumonia, within 10 days prior to inclusion
- Positive result of SARS-CoV-2 PCR test, or any emerging and validated diagnostic laboratory test for COVID-19, within 15 days prior to inclusion
- Under mechanical ventilation administered through an endotracheal tube, for no more than 5 days
- prior Clinical Frailty Scale < 6
- written consent of the patient, or – if impossible - of a relative acting as the legal representative, or – if impossible - of a physician from a non-participating department of the same hospital acting as an impartial witness (see trial procedures)
- No pregnancy
- No prior episode of transfusion-related side effect
- No medical decision to limit therapy
- No current participation in another trial testing a COVID-19 therapy

9.5.4.2 Inclusion visit (Day 1)

Parameters will be obtained as part of the routine clinical care. They will include

- Date of Birth
- Male/Female
- Weight kg
- Height cm
- Clinical frailty scale (appendix F)
- ADL (estimated before COVID-19)
- EQ-5D-5L (estimated before COVID-19)
- marital status
- highest diploma
- work situation
- Reference serum creatinine

Comorbidities (yes/no)

- Hypertension
- Coronary heart disease
- Congestive heart failures

- Chronic obstructive pulmonary diseases
- Asthma
- Fibrotic pulmonary disease
- Neurological disease
- Connective tissue disease
- Diabetes
- Chronic renal failure
- Cirrhosis
- Active solid cancer without metastases
- Active solid cancer with metastases
- Haematological cancer
- AIDS/HIV

Recent medications within the last 3 months

- Steroids
- Immunotherapy
- Anti-cancer chemotherapy
- Immunosuppressive drugs

Current hospitalisation

- Date of hospital admission
- Date of Covid infection diagnosis
- Date of ICU admission
- Date of Intubation
- Date of study inclusion

Clinical findings

- APACHE II score calculation, based on the following criteria
- SOFA score calculation, based on the following criteria
- Glasgow coma score
- Systolic arterial pressure
- Mean arterial pressure
- Need of vasopressor
- Need of ECMO
- Peep
- Pplat
- Prone positioning

- 24 h diuresis
- Need of renal replacement therapy
- Highest temperature

Biologic findings

- Haemoglobin
- Haematocrit
- Platelet count
- White blood cells count
- Neutrophil
- Lymphocyte
- PaO2
- FiO2
- Na
- K
- urea
- creatinin
- protein
- bilirubin
- ferritin
- CRP
- Ddimer
- fibrinogen
- LDH
- TGO
- TGP
- CPK
- Time of administration of the convalescent plasma (in days from disease onset)
- Number, volume and anti-body titre of transfused units
- Any serious adverse reactions or events possibly linked to the transfusion
- WHO progression scale
- Total and neutralizing antibodies to SARS-CoV-2 (centralized)

9.5.4.3 Assessments visits D7 (+-1 day)

- SOFA score calculation
- Glasgow coma score

- Peep level
- Plateau pressure
- tidal Volume
- Respiratory rate
- FiO2
- PaO2
- PaCO2
- Mean arterial pressure
- Inotropic or vasopressor support for at least one hour
    - Dopamine,
    - dobutamine,
    - noradrenaline < 0,1µg/kg/min
    - noradrenaline > 0,1 µg/kg/min,
    - adrenaline <0,1 µg/kg/min,
    - adrenaline > 0,1µg/kg/min,
    - vasopressin/-analogues

- Creatinine
- 24 h diuresis
- Platelet count
- Bilirubin
- CRP
- WBC count
- Ferritin
- Lymphocyte count
- Tracheal aspirate for SARS-CoV-2 viral load, expressed as cycle threshold, assessment
- WHO progression scale
- Total and neutralizing antibodies to SARS-CoV-2 (centralized)

9.5.4.4 Assessement visit D14 (+-1 day)

- SOFA score calculation
- Glasgow coma score
- Peep level
- Plateau pressure
- tidal Volume
- Respiratory rate

- FiO2
- PaO2
- PaCO2
- Mean arterial pressure
- Inotropic or vasopressor support for at least one hour
    - Dopamin,
    - dobutamin,
    - noradrenalin < 0,1µg/kg/min
    - noradrenalin > 0,1 ùg/kg/min,
    - adrenalin <0,1 µg/kg/min,
    - adrenalin > 0,1µg/kg/min,
    - vasopressin/-analogues

- Creatinine
- 24 h diuresis
- Platelet count
- Bilirubin
- CRP
- WBC count
- Ferritin
- Lymphocyte count
- Tracheal aspirate for SARS-CoV-2 viral load, expressed as cycle threshold, assessment

9.5.4.5 Assessement visit D21 (+-1 day)

- SOFA score calculation
- Glasgow coma score
- Peep level
- Plateau pressure
- tidal Volume
- Respiratory rate
- FiO2
- PaO2
- PaCO2
- Mean arterial pressure
- Inotropic or vasopressor support for at least one hour

    - Dopamin,
    - dobutamin,
    - noradrenalin < 0,1µg/kg/min
    - noradrenalin > 0,1 ùg/kg/min,
    - adrenalin <0,1 µg/kg/min,
    - adrenalin > 0,1µg/kg/min,
    - vasopressin/-analogues

- Creatinine
- 24 h diuresis
- Platelet count
- Bilirubin
- CRP
- WBC count
- Ferritin
- Lymphocyte count
- Tracheal aspirate for SARS-CoV-2 viral load, expressed as cycle threshold, assessment

9.5.4.6 Assessment visit D28 (+-1 day)

- SOFA score calculation
- Glasgow coma score
- Peep level
- Plateau pressure
- tidal Volume
- Respiratory rate
- FiO2
- PaO2
- PaCO2
- Mean arterial pressure
- Inotropic or vasopressor support for at least one hour
    - Dopamin,
    - dobutamin,
    - noradrenalin < 0,1µg/kg/min
    - noradrenalin > 0,1 µg/kg/min,
    - adrenalin <0,1 µg/kg/min,
    - adrenalin > 0,1µg/kg/min,

    - vasopressin/-analogues
- Creatinin
- 24 h diuresis
- Platelet count
- Bilirubin
- CRP
- WBC count
- Ferritin
- Lymphocyte count
- Tracheal aspirate for SARS-CoV-2 viral load, expressed as cycle threshold, assessment
- Total and neutralizing antibodies to SARS-CoV-2 (centralized)

9.5.4.7 ICU discharge

- Date of ICU discharge
- WHO progression scale

9.5.4.8 Assessment visit Day 90 (+-15 days)

- Vital status (dead/alive)
- Body weight
- Return to work (Y/N)
- ADL scale (appendix C)
- Anxiety depression scale (appendix D)
- EQ-5D-5L scale (appendix E)
- Total and neutralizing antibodies to SARS-CoV-2 (centralized)

9.5.4.9 Assessment visit Day 365 (+- 30 days)

- Vital status (dead/alive)
- Body weight
- Return to work (Y/N)
- ADL scale

- Anxiety depression scale
- EQ-5D-5L scale

This visit will be performed by phone call or by courier.

9.5.4.10 Discharge from the hospital

- Date of ICU discharge
- Patient alive (Y/N)
  - If Yes :
    - Date of last extubation
    - Destination (Home, revalidation, acute care unit)
-
- Tracheostomy during the ICU stay
  - Y/N
  - date

- Date of hospital discharge

9.5.5 Data to be shared at the European level regarding convalescent plasma use

https://ec.europa.eu/health/blood_tissues_organs/covid-19_en

Register:

https://ec.europa.eu/health/sites/health/files/blood_tissues_organs/docs/guidance_plasma_covid19_en.pdf

- Gender, age range (30-39, 40-49 etc.), co-morbidities
- Transfusion time point (in days from disease onset)
- Number, volume and anti-body titre of transfused unit(s
- Therapies administered to the patient in parallel (other than supportive care)
- Clinical symptoms and laboratory parameters– according to the disease progression scale (Appendix B) at the following time points:
  - Prior to transfusion

- 5 days after transfusion
- At discharge (if the patient survives)

- Any serious adverse reactions or events possibly linked to the transfusion
- Length of hospitalisation (if no death).

9.5.12 Assessment visits in case of discharge before day 90

In case of acute care hospital discharge before day 90, the day 90 visit will be performed by phone call.

9.5.13 Laboratory tests

The laboratory tests collected are based on the descriptive series of critically ill patients already published on COVID-19, mostly from China and Italy, and on the usual tests collected during the treatment of acute respiratory distress syndrome (ARDS) associated with infection or sepsis.

9.5.14 Premature discontinuation of trial treatment

The treatment will be delivered as a single administration of convalescent plasma. In case of severe adverse reaction during the administration (specifically hemodynamic shock), the study plasma will be stopped, and the adverse event described and collected.

If for any reason the treatment is either not administered or prematurely stopped, the patient will remain in the trial for the intention to treat analysis.

No cross-over will be allowed.

Procedures*	Visits								
	Screening	Inclusion	Treatment Phase				Follow Up		
		D1	D7	D14	D21	D28	ICU Discharge	D90	D365
Informed consent		x							
Eligibility assessment	x	x							
Randomisation		x							
Medical history<br>Comorbidities<br>Prior medications		x							
APACH II score		x							
SOFA score		x	x	x	x	x			
WHO disease progression scale		x	x				x		
CRP<br>Viral load (NP or tracheal PCR)		x	x	x	x	x			
Clinical frailty scale		x							
Concomitant drugs against SARS-CoV-2	x	x	x	x	x	x			
Biological findings		x *a	x *b	x *b	x *b	x *b			
24h-diuresis		x	x	x	x	x			
Serum sample for central analysis		x	x			x		x	
Mechanical ventilation		x	x	x	x	x			
Renal replacement therapy		x	x	x	x	x			

Vasoactive drugs		x	x	x	x	x			
ECMO		x	x	x	x	x			
Nosocomial infections		x	x	x	x	x			
Iatrogenic events		x	x	x	x	x			
Adverse events & Serious adverse event		x	x	x	x	x			
ADL scale		x						x	x
Anxiety-depression scale								x	x
EQ-5D-5L scale		x						x	x

* Please see section 9.5 for more details

a. Haemoglobin, Haematocrit, Platelet count, White blood cells count, Neutrophil, Lymphocyte, PaO2, FiO2, Na, K, urea, creatinin, protein, bilirubin, ferritin, CRP, Ddimer, fibrinogen, LDH, TGO, TGP, CPK
b. creatinin, platelet count, bilirubin, CRP, WBC count, Ferritin, Lymphocyt count

9.6. Efficacy and Safety Variables

9.6.1 Efficacy and Safety Measurements Assessed and Flow chart

Measurements will be performed by the investigators and clinical research assistants during their stay in the hospital and during medical visits at each time of endpoints (days 7, 14, 28, 90) in case of hospital discharge, and by phone for day 365 endpoint.

Vital status, dependence on the ventilator and clinical and biological data to calculate the SOFA score will be collected from the patient's record. Viral load, expressed as cycle threshold, will be assessed from tracheal aspirates or naso-pharyngeal swabs and RT-PCR assessment by the usual technique of the routine laboratory. Adverse events possibly related to study intervention will be collected prospectively, according to definitions of iatrogenic events, transfusion-related events and nosocomial infections by the investigator and the clinical research assistant in charge of the study. Scales assessing functional state, psychological state and quality of life will be collected at day 90 visit and over the phone at day 365 visit by a clinical research assistant dedicated to this task by the coordinating center. The adverse events will be collected and severity rating according to Belgian guidelines.

9.6.2 Appropriateness of Measurements

All the efficacy or safety assessments will be standard and consistent with international guidelines.

9.6.3 Primary Efficacy Variable

The primary endpoint used to determine efficacy will be mortality at day 28.

9.7. Safety Reporting

- Adverse events possibly related to study intervention
- SAEs.
- Transfusion related reactions. Transfusion of plasma is procedure with a long-standing experience. The only safety concern with convalescent plasma during acute hospital are transfusion related side effects like transfusion related acute lung injury, serious allergic transfusion reactions and transfusion associated circulatory overload.

9.7.1 Definitions

- An adverse event (AE) is any new, undesirable medical experience or change of an existing condition which occurs during or after treatment, whether considered related to the treatment received. Adverse events will be graded according to the NCI Common Terminology Criteria for Adverse Events (CTCAE) v4.03 as: 1=mild, 2=moderate, 3=severe, 4=life threatening, and 5=fatal. Relatedness to the plasma therapy will be scored as follows: 1=not related, 2=unlikely related, 3=possibly related, 4=probably related and 5=definitely related.

- A serious adverse event (SAE) is any experience with a significant hazard and includes any event that:
  - is fatal
  - is life-threatening (places the patient at immediate risk of death)
  - requires or prolongs hospitalization
  - is permanently or significantly disabling/incapably
  - is a (new) malignancy
  - is a congenital anomaly/birth defect
  - is a known or suspected overdose

9.7.2 Safety reporting procedures

AEs at least possibly related to the plasma infused are to be reported in the CRF.

Any AE should be documented and followed until it is resolved. New AE appearing after the start of a subsequent disease treatment that is not part of this protocol should not be reported anymore.

Adverse events (as specified above) and Serious adverse events must be reported from the initiation of treatment until day 28 after treatment, excepted death that has to be reported until the primary endpoint is reached.

SAEs must be recorded on the Serious Adverse Event Report form, and reported by e-mail to sponsor within one working day (24 hours) of discovery. The local investigator is responsible for transmission to the Principal Investigator and to the chair of the DSMB.

9.8. Confidentiality and data handling

- Data will be collected at each center on the eCRF. The investigator of each participating center will be responsible for entering these data into the central database with the help of a clinical research assistant. All statistical analyses will be performed centrally in Liège.
- Patient data will remain confidential, but paper and/or electronic medical records may be reviewed for trial purposes by authorized individuals other than the treating physicians that are involved in the conduct of the trial.All patients are assigned a unique patient number. This number will be used in any publication that describes the research that the patient has participated in, thereby preventing identification of the patient. All research records are maintained in a locked room, and access to electronic files are restricted by a password. Data protection according to the EU General Data Protection Regulation (2016/79 of April 27th, 2016) is described in details in each site agreement (Data protection agreement)

9.9. Statistical Analysis

- Primary outcome

The primary outcome of the trial is mortality at day 28. Based on prior reports from China, Italy and UK, and consistent with personal observations, we anticipate that the mortality at day 28 will be around 40 %. As no comparative study has yet been published, the reduction that we anticipate is empiric. We consider that a one third relative reduction in 28-day mortality is realistic and clinically relevant. As this reduction might be greater, we plan to perform an interim analysis for ethical reasons. With a two-sided α-risk of 0.05 and a β-risk of 0.20, the minimal number of patients to include to reach significance for a one third relative reduction in 28-day mortality (an absolute reduction of 13.5% day-28 mortality in the intervention group assuming a day-28 mortality of 40 % in the control group) is 250 in the control group and 250 in the active group (total number of patients = 500). This number allows for four interim analyses to be performed in addition to the final analysis, respectively after assessment of 100, 200, 300, 400 and 500 patients. Considering that others trials in COVID-19 may result in modification in mortality and therefore in standard of care, we anticipate that the number of patients that is necessary to address our hypothesis may vary, and will have to be adapted depending on the results of the interim analyses.

- Statistical analysis plan

Results will be analysed in intention to treat and expressed as means and standard deviations (SD) for quantitative variables with a Normal distribution and as medians and 25th to 75th percentiles for skewed distributed quantitative variables. Qualitative variables will be expressed using counts and percentages.

Mean values between groups will be compared by Student t-test but also by the non-parametric Wilcoxon test when normality assumption is not fulfilled. Proportions will be compared by the chi-square

test. Appropriate multivariate analyses will be performed to assess the relationship between outcome and patients' characteristics and clinical features.

For the primary endpoints, vital status at day 28 in all groups will be calculated with corresponding 95% CI. Comparison between groups will be tested using chi-square test.

Regarding the secondary endpoints, quantitative values between groups will be compared by Student t-test or non-parametric Wilcoxon for the SOFA score (at different time points), viral load of SARS-CoV-2 (at different time points), circulating CRP (at different time points), ADL score at day 90, anxiety-depression scale at day 90, sero-conversion. Poisson regression analysis will be considered to analyse number of ventilator-free days up to day 28. Comparison of proportions will be applied to compare location of patient at day 90, as well as presence of various adverse events. If descriptive analysis revealed the presence of potential confounding factors, appropriate multivariate analysis will then be considered.

The two-sided level of significance used in this trial is 5%. Statistical analyses will be carried out using SAS (version 9.4 for Windows) statistical package.

- Interim analysis plan

Due to ethical considerations of the current situation, we propose to conduct a group-sequential design with several interim analyses. The objective of including interim analyses in the study design is to allow the possibility of stopping the study early if a clinically relevant benefit is already established from a statistical point of view. Conversely, the interim analyses also allow the possibility of an early stopping of the study if the observed benefit, at the time of the interim analysis, is so small as to make it extremely unlikely that the study would succeed, were it continued until full accrual. Finally, the interim analyses will also include a futility stopping rule for the SOC group, which may appear to be unjustified if mortality in that group appears too large to be acceptable.

The interim analyses will be performed with 100, 200, 300 and 400 patients evaluable for 28-day mortality. Two Lan-Demets spending functions will be used: one, similar to a Pocock (aggressive) boundary to stop the study for futility, and the other, similar to an O'Brien-Fleming (conservative) boundary to stop the study for extreme efficacy, If the interim analyses take place with increments of exactly 100 patients, and assuming a mortality of 40% in the control group, the recommendations to stop the study for futility or for efficacy are summarized in Table 1. Of note, the trial will reach statistical significance if the absolute difference in 28-day mortality is 9% or larger, which might still be a clinically worthwhile difference. The rules for DSMB recommendations shown in Table 1 will not be binding.

Table 1: *interim analyses and potential recommendations of the DSMB to stop the trial for futility or efficacy, depending on the observed treatment effect (and associated P-value)*

Interim analysis	Number of patients	Recommend stopping for futility if difference in mortality is smaller than	Recommend stopping for efficacy if difference in mortality is greater than
1	100	0.02 (P > 0.83)	-
2	200	0.04 (P > 0.60)	0.22 (P < 0.001)
3	300	0.06 (P > 0.28)	0.14 (P < 0.007)
4	400	0.08 (P > 0.12)	0.11 (P < 0.022)
5 (final)	500	-	0.09 (P < 0.042)

9.10. Changes in the Conduct of the Study or Planned Analyses

- Provision for adaptive design changes at the time of interim analyses

Because of the considerable uncertainties about the outcomes that will be observed in the two arms of the trial, the DSMB will be allowed to recommend design changes based on the observed data at the times of the interim analyses. These changes may include an adaptation of the sample size or the patient selection. Any such changes are adaptive in nature and will be implemented using appropriate methods to control the impact of these changes on the operating characteristics of the trial (especially the probability of type I error).

9.11. Protocol Amendements

If amendments to the protocol (modifying sense or objectives or modifying the undergone constraints or the risks incurred by the subjects) turn out to be necessary, they will be submitted to the opinion of the EC having examined the initial protocol.

10. Study patients

10.1. Disposition of patient

- Clear accounting of all patients who entered the study (e.g. randomized, completed the study, screened, discontinued, …)
- Give reasons for discontinuation
- State whether blind was broken

10.2. Protocol Deviations

All deviations related to study inclusion or exclusion criteria, conduct of the trial, patient management or patient assessment should be described.

10.3. Data Management Responsibilities

The study will be monitored by a DSMB that will include international members not involved in this protocol and a statistician. The DSMB will review the safety data at least every 6 months and at each interim analysis.

10.4. Study Data, Data Ownership and Data Sharing with KCE

After the completion of the study the Sponsor will transfer the pseudonymized study data set to KCE. KCE will request approval from the competent chamber of the Information Security Committee to have the relevant study data linked with IMA data by a trusted third party (TTP, eHealth platform) using the patient national number.

The patient information and consent includes wording that the **national number** will be recorded on site by the investigator for later data linkage. The patient information and consent will also include that in case the patient is randomized, it is planned that a trusted third party (TTP, eHealth platform) will receive and use the national number to link with IMA administrative data. This data linkage is planned to obtain a more complete data set that will be used for the analysis of effectiveness and cost-effectiveness of the intervention by KCE.

KCE and Sponsor have entered into a research agreement detailing the roles and responsibilities of each party, as well as other legal aspects of this collaboration, including the right to use and access of KCE to the Study Data.

"Background" means any intellectual property (IP), data, materials, information owned or controlled by the Sponsor or a Site, and required to run this Study. Sponsor will identify such Background including the legal restrictions of which Sponsor or Sites are aware that may affect the use of the Background for the purpose of the Study or the rights granted to KCE under this Agreement.

The Study Data consist of this protocol, including amendments, the electronic forms for data capture, including the annotations and guidance for use, the electronic database of the pseudonymized clinical and non-clinical data collected using data capture, including the log of changes from data entry to

database lock, study reports based on these pseudonymized data, and any data or reports generated at a later stage, eg based on exploratory analyses or stored samples.

"Foreground" means any Study Data, and any tangible biological, chemical and physical material and inventions, that are generated, acquired, discovered, conceived, developed, created, exemplified or derived as a result of carrying out the Clinical Study, whatever its form or nature, whether it can be protected or not, as well as any Foreground IP. Sponsor acknowledges that the main purpose of the research performed under this Agreement is to generate results that will serve the general public interests, and specifically the interests of the patients and public healthcare decision making bodies, and, therefore, undertakes not to exploit the Foreground in any way that is or could be detrimental to such interests.

The Sponsor owns the Study Data, but provides KCE with a copy of the pseudonymized database after database lock as well as a royalty-free unrestricted license to use the Study Data for non-commercial public health related purposes as detailed in the Agreement between KCE and Sponsor. If judged appropriate, KCE will introduce the request to the competent chamber of the Information Security Committee and arrange for the data linkage. For the sake of clarity, the linked data are not part of the Study Data. However, KCE will discuss with the Sponsor the results of the analyses and the reporting of the linked data.

11. Finance and Insurance

- Insurance without fault (Law of 7 May 2004) : Ethias Insurance

  According to the Belgian law, the principal investigator is responsible for any damage, even without fault, caused directly or indirectly to the participant by the experimentation. Therefore, a specific trial insurance will be provided for patients treated in Belgium through the CHU of Liège.

- The financial agreement between the promotor, the investigator and the Institution, incorporates:
  - Data protection
  - Conflict of interests

12. Dissemination of Results and Publication Policy

The results will be submitted for presentation to international societies of critical care and/or infectious diseases and for publication to an international scientific medical journal. The authorship will include the members of the writing and/or steering committee, the investigators from centers having recruited at least 10 patients, a representative of the Belgian Red Cross and transfusion services, the biostatistician of the study and all scientists whose contribution will have been substantial. Pseudonymised Data from this study can be used by the funder (KCE) (for more details, please check the research agreement signed between sponsor and KCE) or similar public healthcare research institutes in Europe for further analyses, for example to determine whether one of the treatments studied provides better value in meta-analyses.

The data required by the European requirements will be shared according to https://ec.europa.eu/health/blood_tissues_organs/covid-19_en, and the trial will be registered at https://ec.europa.eu/health/sites/health/files/blood_tissues_organs/docs/guidance_plasma_covid19_en.pdf.

13. Archiving

The CHU de Liège, more specifically the Department of Intensive Care provides a secure locked office for the archiving of all study documents related to the study as in the Trial Master File (CRF, ICF, Source documents,…) during at least 25 years (as to Circular 536/2014, Art.58).

As to the Data Processing Agreement:

Patient data will be collected by personnel of the clinical department where patient is taken care of, all members of hospital staff.

Data will be stored in electronic format in the main database at CHU de Liège for 25 years, Department of Intensive Care.

Informed consent forms signed by patient (or legal representative or impartial witness) and physician, as well as all trial documentation, be it in paper or electronic format, shall be stored in the clinical department where patient is taken care of in a secure, locked office. A copy of the signed consent form will also be scanned in the electronic patient medical file of the respective hospital.

All trial documentation communicated to the sponsor, be it in paper or electronic format, shall be stored in the Department of Intensive Care at CHU de Liège in a secure, locked office.

14. Study Report

The final report will be written after statistical analyses and interpretation, preferably within 3 months after the last visit of the last patient. The submission for publication of the main results should be available within one month after day 28 of the last patient included. This short schedule is motivated by the importance of the global crisis induced by the COVID-19 pandemic.

15. Literature References

1. Vincent JL, de Mendonça A, Cantraine F, Moreno R, Takala J, Suter PM, et al. Use of the SOFA score to assess the incidence of organ dysfunction/failure in intensive care units: results of a multicenter, prospective study. Working group on “sepsis-related problems” of the European Society of Intensive Care Medicine. Crit Care Med. 1998;26:1793–800.

2. To KK-W, Tsang OT-Y, Leung W-S, Tam AR, Wu T-C, Lung DC, et al. Temporal profiles of viral load in posterior oropharyngeal saliva samples and serum antibody responses during infection by SARS-CoV-2: an observational cohort study. Lancet Infect Dis. 2020;

3. Katz S, Ford AB, Moskowitz RW, Jackson BA, Jaffe MW. STUDIES OF ILLNESS IN THE AGED. THE INDEX OF ADL: A STANDARDIZED MEASURE OF BIOLOGICAL AND PSYCHOSOCIAL FUNCTION. JAMA. 1963;185:914–9.

4. Zigmond AS, Snaith RP. The hospital anxiety and depression scale. Acta Psychiatr Scand. 1983;67:361–70.

5. Herdman M, Gudex C, Lloyd A, Janssen M, Kind P, Parkin D, et al. Development and preliminary testing of the new five-level version of EQ-5D (EQ-5D-5L). Qual Life Res. 2011;20:1727–36.

6. Tabah A, Koulenti D, Laupland K, Misset B, Valles J, Bruzzi de Carvalho F, et al. Characteristics and determinants of outcome of hospital-acquired bloodstream infections in intensive care units: the EUROBACT International Cohort Study. Intensive Care Med. 2012;38:1930–45.

7. Girard R, Baboi L, Ayzac L, Richard J-C, Guérin C, Proseva trial group. The impact of patient positioning on pressure ulcers in patients with severe ARDS: results from a multicentre randomised controlled trial on prone positioning. Intensive Care Med. 2014;40:397–403.

8. Anzueto A, Frutos-Vivar F, Esteban A, Alía I, Brochard L, Stewart T, et al. Incidence, risk factors and outcome of barotrauma in mechanically ventilated patients. Intensive Care Med. 2004;30:612–9.

9. Garrouste-Orgeas M, Soufir L, Tabah A, Schwebel C, Vesin A, Adrie C, et al. A multifaceted program for improving quality of care in intensive care units: IATROREF study. Crit Care Med. 2012;40:468–76.

10. Coronavirus COVID-19 (2019-nCoV) [Internet]. [cited 2020 Apr 12]. Available from: https://gisanddata.maps.arcgis.com/apps/opsdashboard/index.html#/bda7594740fd40299423467b48e9ecf6

11. Epistat – Covid-19 [Internet]. [cited 2020 Apr 12]. Available from: https://epistat.wiv-isp.be/covid/covid-19.html

12. Emanuel EJ, Persad G, Upshur R, Thome B, Parker M, Glickman A, et al. Fair Allocation of Scarce Medical Resources in the Time of Covid-19. N Engl J Med. 2020;

13. ICNARC – Reports [Internet]. [cited 2020 Apr 12]. Available from: https://www.icnarc.org/Our-Audit/Audits/Cmp/Reports

14. Wu C, Chen X, Cai Y, Xia J, Zhou X, Xu S, et al. Risk Factors Associated With Acute Respiratory Distress Syndrome and Death in Patients With Coronavirus Disease 2019 Pneumonia in Wuhan, China. JAMA Intern Med. 2020;

15. Guérin C, Reignier J, Richard J-C, Beuret P, Gacouin A, Boulain T, et al. Prone positioning in severe acute respiratory distress syndrome. N Engl J Med. 2013;368:2159–68.

16. Zhao J, Yuan Q, Wang H, Liu W, Liao X, Su Y, et al. Antibody responses to SARS-CoV-2 in patients of novel coronavirus disease 2019. Clin Infect Dis. 2020;

17. Li H, Zhou Y, Zhang M, Wang H, Zhao Q, Liu J. Updated approaches against SARS-CoV-2. Antimicrob Agents Chemother. 2020;

18. Le TT, Andreadakis Z, Kumar A, Román RG, Tollefsen S, Saville M, et al. The COVID-19 vaccine development landscape. Nature Reviews Drug Discovery [Internet]. 2020 [cited 2020 Apr 12]; Available from: https://www.nature.com/articles/d41573-020-00073-5

19. Shen C, Wang Z, Zhao F, Yang Y, Li J, Yuan J, et al. Treatment of 5 Critically Ill Patients With COVID-19 With Convalescent Plasma. JAMA. 2020;

20. Duan K, Liu B, Li C, Zhang H, Yu T, Qu J, et al. Effectiveness of convalescent plasma therapy in severe COVID-19 patients. Proc Natl Acad Sci USA. 2020;

21. Cheng Y, Wong R, Soo YOY, Wong WS, Lee CK, Ng MHL, et al. Use of convalescent plasma therapy in SARS patients in Hong Kong. Eur J Clin Microbiol Infect Dis. 2005;24:44–6.

22. Mair-Jenkins J, Saavedra-Campos M, Baillie JK, Cleary P, Khaw F-M, Lim WS, et al. The effectiveness of convalescent plasma and hyperimmune immunoglobulin for the treatment of severe acute respiratory infections of viral etiology: a systematic review and exploratory meta-analysis. J Infect Dis. 2015;211:80–90.

23. Maddur MS, Othy S, Hegde P, Vani J, Lacroix-Desmazes S, Bayry J, et al. Immunomodulation by intravenous immunoglobulin: role of regulatory T cells. J Clin Immunol. 2010;30 Suppl 1:S4-8.

24. Campbell CM, Kahwash R. Will Complement Inhibition be the New Target in Treating COVID-19 Related Systemic Thrombosis? Circulation. 2020;

25. Hanff TC, Harhay MO, Brown TS, Cohen JB, Mohareb AM. Is There an Association Between COVID-19 Mortality and the Renin-Angiotensin System-a Call for Epidemiologic Investigations. Clin Infect Dis. 2020;

26. Lew RA, Warner FJ, Hanchapola I, Yarski MA, Ramchand J, Manohar J, et al. Angiotensin-converting enzyme 2 catalytic activity in human plasma is masked by an endogenous inhibitor. Exp Physiol. 2008;93:685–93.

27. Toy P. Update on transfusion-related acute lung injury. Clin Adv Hematol Oncol. 2019;17:378–81.

28. Wan Y, Shang J, Sun S, Tai W, Chen J, Geng Q, et al. Molecular Mechanism for Antibody-Dependent Enhancement of Coronavirus Entry. J Virol. 2020;94.

29. Yang KD, Yeh WT, Yang MY, Chen RF, Shaio MF. Antibody-dependent enhancement of heterotypic dengue infections involved in suppression of IFNgamma production. J Med Virol. 2001;63:150–7.

30. Joyner MJ, Wright RS, Fairweather D, Senefeld JW, Bruno KA, Klassen SA, et al. Early safety indicators of COVID-19 convalescent plasma in 5,000 patients. J Clin Invest. 2020;

31. Wu F, Liu M, Wang A, et al. Evaluating the Association of Clinical Characteristics With Neutralizing Antibody Levels in Patients Who Have Recovered From Mild COVID-19 in Shanghai,

China [published online ahead of print, 2020 Aug 18]. JAMA Intern Med. 2020;10.1001/jamainternmed.2020.4616. doi:10.1001/jamainternmed.2020.4616 .

32. Guidet B, de Lange DW, Boumendil A, Leaver S, Watson X, Boulanger C, et al. The contribution of frailty, cognition, activity of daily life and comorbidities on outcome in acutely admitted patients over 80 years in European ICUs: the VIP2 study. Intensive Care Med. 2020;46:57–69.

16. Appendix

A. SOFA score

System	Score				
	0	1	2	3	4
Respiration					
PaO_2/FIO_2, mmHg (kPa)	≥400 (53.3)	<400 (53.3)	<300 (40)	<200 (26.7) with respiratory support	<100 (13.3) with respiratory support
Coagulation					
Platelets, $\times 10^3$ μL^{-1}	≥150	<150	<100	<50	<20
Liver					
Bilirubin, mg dL^{-1} (μmol L^{-1})	<1.2 (20)	1.2–1.9 (20–32)	2.0–5.9 (33–101)	6.0–11.9 (102–204)	>12.0 (204)
Cardiovascular	MAP ≥ 70 mmHg	MAP < 70 mmHg	Dopamine < 5 or dobutamine (any dose)[a]	Dopamine 5.1–15 or epinephrine ≤ 0.1 or norepinephrine ≤ 0.1[a]	Dopamine > 15 or epinephrine > 0.1 or norepinephrine > 0.1[a]
Central Nervous System (CNS)					
Glasgow Coma Scale score[b]	15	13–14	10–12	6–9	<6
Renal					
Creatinine, mg dL^{-1} (μmol L^{-1})	<1.2 (110)	1.2–1.9 (110–170)	2.0–3.4 (171–299)	3.5–4.9 (300–440)	>5.0 (440)
Urine output, mL per day				<500	<200

FIO_2: fraction of inspired oxygen; MAP: mean arterial pressure; PaO_2: partial pressure of oxygen.
[a]Catecholamine doses are given as μg kg^{-1} min^{-1} for at least 1 h.
[b]Glasgow Coma Scale scores range from 3 to 15; higher score indicates better neurological function.

B. WHO progression scale

OMS Progression scale	Descriptor	Score
Uninfected	Uninfected; non viral RNA detected	0
Ambulatory	Asymptomatic; viral RNA detected	1
Ambulatory	Symptomatic; Independent	2
Ambulatory	Symptomatic; Assistance needed	3
Hospitalized : mild disease	Hospitalized; No oxygen therapy	4
Hospitalized : mild disease	Hospitalized; oxygen by mask or nasal prongs	5
Hospitalized : severe disease	Hospitalized; oxygen by NIV or High flow	6
Hospitalized : severe disease	Intubation and Mechanical ventilation, pO2/FIO2>=150 OR SpO2/FIO2>=200	7
Hospitalized : severe disease	Mechanical ventilation, (pO2/FIO2<150 OR pO2/FIO2<200) OR vasopressors (norepinephrine >0.3 microg/kg/min)	8
Hospitalized : severe disease	Mechanical ventilation, pO2/FIO2<150 AND vasopressors (norepinephrine >0.3 microg/kg/min), OR Dialysis OR ECMO	9
Death	Dead	10

C. Katz Index of Independence in Activities of Daily Living

ACTIVITIES	INDEPENDENCE:	DEPENDENCE:
POINTS (1 OR 0)	(1 POINT)	(0 POINTS)
	**NO** supervision, direction or personal assistance	**WITH** supervision, direction, personal assistance or total care
**BATHING**<br>POINTS:___________	(1 POINT) Bathes self completely or needs help in bathing only a single part of the body such as the back, genital area or disabled extremity.	(0 POINTS) Needs help with bathing more than one part of the body, getting in or out of the tub or shower. Requires total bathing.
**DRESSING**<br>POINTS:___________	(1 POINT) Gets clothes from closets and drawers and puts on clothes and outer garments complete with fasteners. May have help tying shoes.	(0 POINTS) Needs help with dressing self or needs to be completely dressed.
**TOILETING**<br>POINTS:___________	(1 POINT) Goes to toilet, gets on and off, arranges clothes, cleans genital area without help.	(0 POINTS) Needs help transferring to the toilet, cleaning self or uses bedpan or commode.
**TRANSFERRING**<br>POINTS:___________	(1 POINT) Moves in and out of bed or chair unassisted. Mechanical transferring aides are acceptable.	(0 POINTS) Needs help in moving from bed to chair or requires a complete transfer.
**CONTINENCE**<br>POINTS:___________	(1 POINT) Exercises complete self control over urination and defecation.	(0 POINTS) Is partially or totally incontinent of bowel or bladder.
**FEEDING**<br>POINTS:___________	(1 POINT) Gets food from plate into mouth without help. Preparation of food may be done by another person.	(0 POINTS) Needs partial or total help with feeding or requires parenteral feeding.

TOTAL POINTS = ______ *6 = High (patient independent) 0 = Low (patient very dependent)*

D. Anxiety depression scale

Hospital Anxiety and Depression Scale (HADS)

Tick the box beside the reply that is closest to how you have been feeling in the past week. Don't take too long over you replies: your immediate is best.

D	A		D	A	
		**I feel tense or 'wound up':**			**I feel as if I am slowed down:**
	3	Most of the time	3		Nearly all the time
	2	A lot of the time	2		Very often
	1	From time to time, occasionally	1		Sometimes
	0	Not at all	0		Not at all
		**I still enjoy the things I used to enjoy:**			**I get a sort of frightened feeling like 'butterflies' in the stomach:**
0		Definitely as much		0	Not at all
1		Not quite so much		1	Occasionally
2		Only a little		2	Quite Often
3		Hardly at all		3	Very Often
		**I get a sort of frightened feeling as if something awful is about to happen:**			**I have lost interest in my appearance:**
	3	Very definitely and quite badly	3		Definitely
	2	Yes, but not too badly	2		I don't take as much care as I should
	1	A little, but it doesn't worry me	1		I may not take quite as much care
	0	Not at all	0		I take just as much care as ever
		**I can laugh and see the funny side of things:**			**I feel restless as I have to be on the move:**
0		As much as I always could		3	Very much indeed
1		Not quite so much now		2	Quite a lot
2		Definitely not so much now		1	Not very much
3		Not at all		0	Not at all
		**Worrying thoughts go through my mind:**			**I look forward with enjoyment to things:**
	3	A great deal of the time	0		As much as I ever did
	2	A lot of the time	1		Rather less than I used to
	1	From time to time, but not too often	2		Definitely less than I used to
	0	Only occasionally	3		Hardly at all
		**I feel cheerful:**			**I get sudden feelings of panic:**
3		Not at all		3	Very often indeed
2		Not often		2	Quite often
1		Sometimes		1	Not very often
0		Most of the time		0	Not at all
		**I can sit at ease and feel relaxed:**			**I can enjoy a good book or radio or TV program:**
	0	Definitely	0		Often
	1	Usually	1		Sometimes
	2	Not Often	2		Not often
	3	Not at all	3		Very seldom

Please check you have answered all the questions

Scoring:

Total score: Depression (D) ___________ Anxiety (A) ____________

0-7 = Normal

8-10 = Borderline abnormal (borderline case)

11-21 = Abnormal (case)

E. EQ-5D

By placing a tick in one box in each group below, please indicate which statements best describe your own health state today.

Mobility

I have no problems in walking about ❑

I have some problems in walking about ❑

I am confined to bed ❑

Self-Care

I have no problems with self-care ❑

I have some problems washing or dressing myself ❑

I am unable to wash or dress myself ❑

Usual Activities (*e.g. work, study, housework, family or leisure activities)*

I have no problems with performing my usual activities ❑

I have some problems with performing my usual activities ❑

I am unable to perform my usual activities ❑

Pain/Discomfort

I have no pain or discomfort ❑

I have moderate pain or discomfort ❑

I have extreme pain or discomfort ❑

Anxiety/Depression

I am not anxious or depressed ❑

I am moderately anxious or depressed ❑

I am extremely anxious or depressed ❑

F. Clinical Frailty Scale

Clinical Frailty Scale*

1 **Very Fit** – People who are robust, active, energetic and motivated. These people commonly exercise regularly. They are among the fittest for their age.

2 **Well** – People who have **no active disease symptoms** but are less fit than category 1. Often, they exercise or are very **active occasionally**, e.g. seasonally.

3 **Managing Well** – People whose **medical problems are well controlled**, but are **not regularly active** beyond routine walking.

4 **Vulnerable** – While **not dependent** on others for daily help, often **symptoms limit activities**. A common complaint is being "slowed up", and/or being tired during the day.

5 **Mildly Frail** – These people often have **more evident slowing**, and need help in **high order IADLs** (finances, transportation, heavy housework, medications). Typically, mild frailty progressively impairs shopping and walking outside alone, meal preparation and housework.

6 **Moderately Frail** – People need help with **all outside activities** and with **keeping house**. Inside, they often have problems with stairs and need **help with bathing** and might need minimal assistance (cuing, standby) with dressing.

7 **Severely Frail** – **Completely dependent for personal care**, from whatever cause (physical or cognitive). Even so, they seem stable and not at high risk of dying (within ~ 6 months).

8 **Very Severely Frail** – Completely dependent, approaching the end of life. Typically, they could not recover even from a minor illness.

9. Terminally Ill - Approaching the end of life. This category applies to people with **a life expectancy <6 months**, who are **not otherwise evidently frail.**

Scoring frailty in people with dementia

The degree of frailty corresponds to the degree of dementia. Common **symptoms in mild dementia** include forgetting the details of a recent event, though still remembering the event itself, repeating the same question/story and social withdrawal.

In **moderate dementia**, recent memory is very impaired, even though they seemingly can remember their past life events well. They can do personal care with prompting.

In **severe dementia**, they cannot do personal care without help.

* 1. Canadian Study on Health & Aging, Revised 2008.
2. K. Rockwood et al. A global clinical measure of fitness and frailty in elderly people. CMAJ 2005;173:489-495.

© 2008 Version 1.2_EN. All rights reserved. Geriatric Medicine Research, Dalhousie University, Halifax, Canada. Permission granted to copy for research and educational purposes only

DALHOUSIE UNIVERSITY Inspiring Minds
